# Mechanisms conferring multi-layered protection against intestinal *Salmonella* Typhimurium infection

**DOI:** 10.1093/femsre/fuaf038

**Published:** 2025-08-12

**Authors:** Sanne Kroon, Wolf-Dietrich Hardt

**Affiliations:** Department of Biology, Institute of Microbiology,ETH Zürich, Zürich, 8093, Switzerland; Department of Biology, Institute of Microbiology,ETH Zürich, Zürich, 8093, Switzerland

**Keywords:** *Salmonella* Typhimurium, Host-pathogen interactions, Microbiota dysbiosis, Intestinal epithelial barrier, Immune defence mechanisms, Infection susceptibility

## Abstract

Enteropathogens cause many gastrointestinal infections every year. However, it is often overlooked that many individuals remain asymptomatic despite exposure to these pathogens. The mechanisms underlying this effective protection against infection may hold important clues for disease prevention or therapy. Here, we focus on *Salmonella enterica* serovar Typhimurium (*S*. Tm), a well-studied enteropathogen closely related to commensal *Escherichia coli*. We discuss the host's multi-layered defence mechanisms that protect against *S*. Tm infection of the intestine, with an emphasis on the microbiota, epithelial barrier, and immune system. Perturbations in these defences, such as microbiota dysbiosis, variability in epithelial barrier integrity, or immune defects, can impair protection and increase susceptibility to disease. Additionally, we review the virulence mechanisms and metabolic adaptations that *S*. Tm has evolved to overcome these protective layers. This complex interplay between host defence layers and pathogen traits, shaped by both intrinsic and extrinsic factors, ultimately determines whether exposure results in asymptomatic carriage or symptomatic disease. Understanding these dynamics is critical for developing targeted interventions to prevent *S*. Tm infections and mitigate their impact on public health.

## Teaser

This review explores the interplay between host defence mechanisms and pathogenic mechanisms employed by *Salmonella* Typhimurium, highlighting how disruptions in the microbiota, epithelial barrier, or immune system influence infection outcomes and inform potential therapeutic interventions.

## Introduction

*Salmonella enterica* serovar Typhimurium (*S*. Tm) is a major cause of gastroenteritis worldwide, a condition characterised by symptoms such as fever, abdominal pain, diarrhoea, nausea, vomiting, weakness and dehydration (World Health Organisation 2015). Exposure to *S*. Tm poses minimal risk to most healthy adults, leading only to transient colonisation in many cases without overt disease symptoms. In contrast, it can more frequently cause severe disease, including bacteraemia, in immunocompromised, pregnant, elderly and hospitalised patients (World Health Organisation 2015). Transmission primarily occurs through ingestion of contaminated food, such as raw or undercooked meat, eggs, or dairy products, as many animals are natural carriers of *S*. Tm (World Health Organisation 2015). Understanding the infection mechanisms of *S*. Tm and how the host's multi-layered defences can prevent disease is crucial to develop effective public health strategies to protect vulnerable populations.

**Figure 1.**
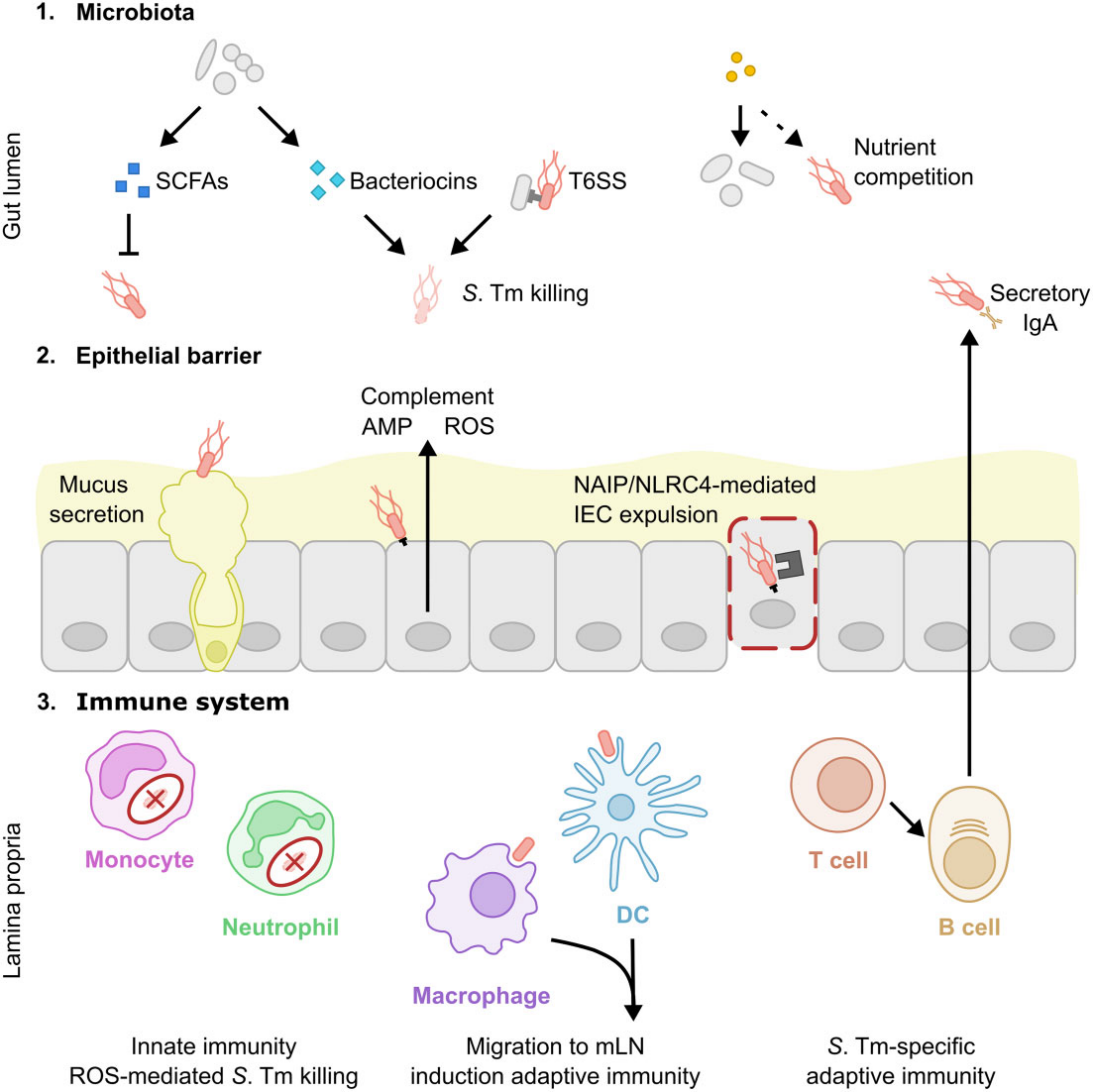
Mechanisms underlying multi-layered protection against intestinal *S*. Tm infection. 1. The microbiota confers colonisation resistance against *S*. Tm, limiting its ability to establish itself in the gut lumen through inhibitory compound production, type 6 secretion system (T6SS) expression and nutrient competition. 2. The mucus and epithelial layer serve as a physical barrier against *S*. Tm invasion. Intestinal epithelial cells (IEC) produce antimicrobial compounds and employ NAIP/NLRC4-mediated expulsion of infected cells to reduce infection of the epithelial barrier. 3. The innate immune system recognises and phagocytoses *S*. Tm, utilising defence mechanisms, such as reactive oxygen species (ROS) production to eliminate *S*. Tm, and migrating to the mesenteric lymph node (mLN) to induce adaptive immune responses. Adaptive immune cells such as T cells and B cells contribute to the protection against *S*. Tm infection through *S*. Tm-specific responses, including *S*. Tm-specific secretory IgA production.

The gastrointestinal (GI) tract is a barrier organ that is constantly exposed to commensal microbiota and ingested food, and also occasionally encounters intestinal pathogens. To defend against pathogenic threats, the gut features multiple layers of protection (Fig. 1). The first layer of protection is provided by the complex microbiota in the intestinal lumen, which competes with incoming bacteria for nutrients, utilises antimicrobial defences and produces inhibitory metabolites, providing colonisation resistance (Woelfel et al. 2024). The second layer of protection consists of the mucus and epithelial layer, which form physical barriers against bacterial translocation into the sterile compartment (Hausmann et al. 2024). Subjacent to the epithelial layer is the lamina propria, a connective tissue rich in immune cells which form a third layer of protection. Innate immune cells, in particular, play a crucial role in detecting and responding to invading pathogens, aiming to eliminate them while preserving tissue homeostasis (Hickey et al. 2023). Additionally, the mesenteric lymph nodes (mLNs) and gut-associated lymphoid tissue (GALT), which include Peyer's patches (PPs), caecal patches and isolated lymphoid follicles (ILFs), are essential for the initiation and regulation of adaptive immune responses (Mörbe et al. 2021). These layers together protect against pathogenic infections and sustain intestinal homeostasis. This makes the GI tract an interesting site to investigate the dynamics between the host, microbiota and pathogens, including *S*. Tm.

**Figure 2.**
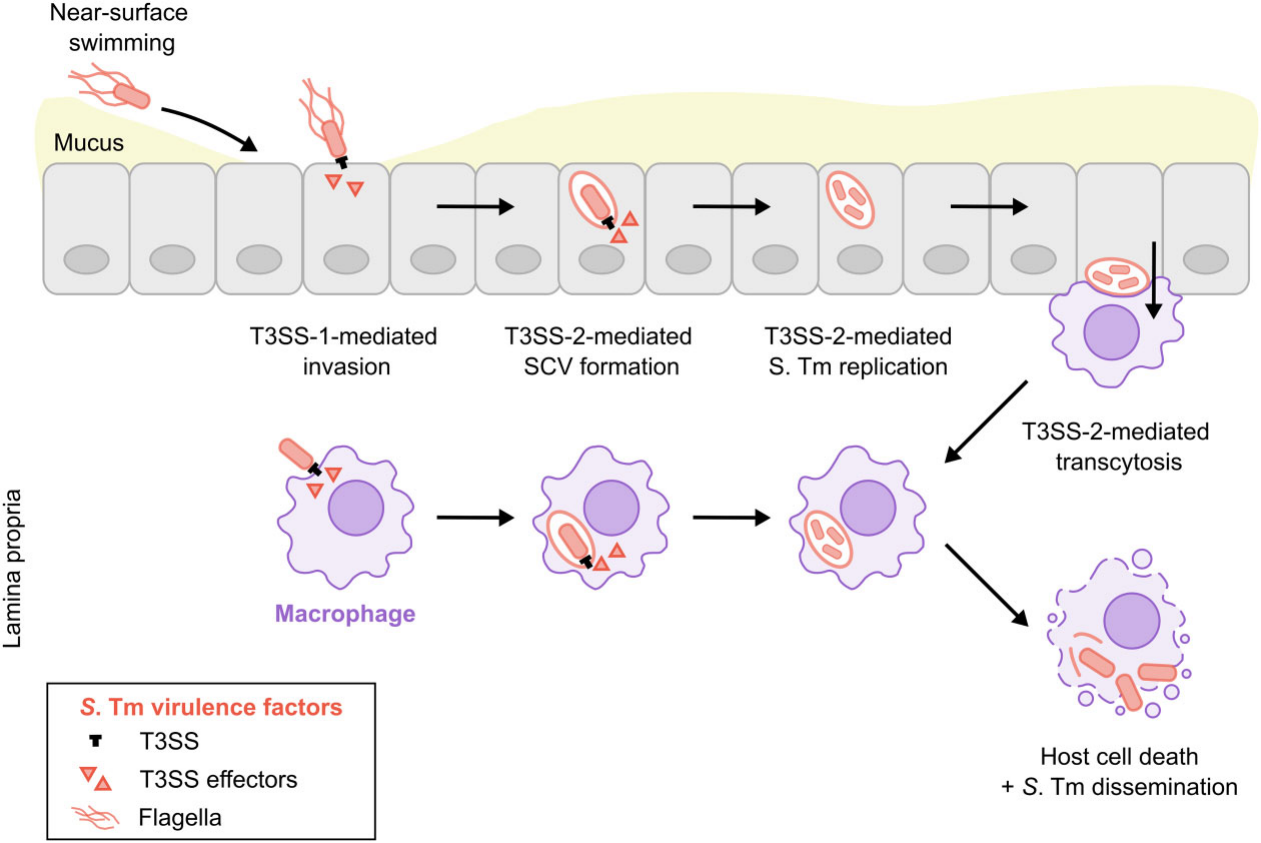
*S*. Tm virulence mechanisms for invasion and replication. *S*. Tm utilises near-surface swimming along the mucus layer to navigate gaps in the mucus layer and reach the epithelium. Then, *S*. Tm uses the T3SS-1 and its effectors to invade IECs and macrophages. Subsequently, *S*. Tm employs the T3SS-2 and its effectors to form a *Salmonella*-containing vacuole (SCV), facilitating intracellular replication. From this niche, *S*. Tm can infect other cells and disseminate systemically.

*S*. Tm has evolved multiple mechanisms to infect a broad range of hosts (Galán 2021). Investigating these mechanisms is fundamental to understand the interplay between *S*. Tm and the host's multi-layered protection, and how *S*. Tm's pathogenic lifestyle contrasts from that of its non-pathogenic *Escherichia coli*-like commensal ancestor (Diard and Hardt 2017). Mouse models have been instrumental for deciphering these mechanisms *in vivo*. While most of the mechanisms discussed in this review will refer to data from mice, we assume *S*. Tm infections in other host species will follow equivalent principles. *S*. Tm employs its flagellum for motility towards nutrient-rich niches and electron acceptors like nitrate, for near-surface swimming along the mucus layer to reach the underlying epithelium, and for adhesion to intestinal epithelial cells (IECs) (Fig. 2)(Stecher et al. 2004, 2008; Misselwitz et al. 2012; Rivera-Chávez et al. 2016a; Furter et al. 2019; Horstmann et al. 2020). Subsequently, *S*. Tm uses virulence factors encoded by *Salmonella* pathogenicity islands 1 and 2 (SPI-1, SPI-2), specifically the type three secretion system 1 and 2 (T3SS-1, T3SS-2), which are essential for host cell invasion and intracellular replication. Through the T3SS-1, *S*. Tm secretes effector proteins, including SipA, SopB, SopE and SopE2, which drive cytoskeleton rearrangement and fosters active invasion into IECs to traverse the epithelial barrier and reach phagocytes in the lamina propria (Hapfelmeier et al. 2004, 2005; Diao et al. 2008; Bueno et al. 2010; Di Martino et al. 2019; Fattinger et al. 2020). Alternatively, luminal *S*. Tm is sampled by conventional dendritic cells (cDCs) or *S*. Tm infects M cells associated with PPs (Hapfelmeier et al. 2008; Niess et al. 2005; Tahoun et al. 2012). Once inside host cells, *S*. Tm employs the T3SS-2 for intracellular replication by forming a *S*. Tm-containing vacuole (SCV) (Garcia-Del Portillo and Finlay 1995; Müller et al. 2012; Laughlin et al. 2014; Chen et al. 2021a). T3SS-2 effectors can modulate SCV formation, interfere with inflammatory signalling and block cell death and autophagy pathways (Pillay et al. 2023). While still not fully understood, this interference with host cell responses is thought to provide *S*. Tm with a niche for replication. In addition, *S*. Tm can disseminate to systemic sites, including mLNs, spleen and liver, where its growth may result in severe disease, particularly in immunocompromised hosts (Galán 2021; Marchello et al. 2021). Overall, *S*. Tm employs multiple virulence factors to overcome the host's multi-layered defence mechanisms and foster infection.

Most of the work describing the mechanisms above has been performed in mouse models with extremely high disease rates, such as the streptomycin mouse model, where 100% of orally infected animals develop mucosal disease within 24 h (Barthel et al. 2003). Interestingly, exposure to *S*. Tm in humans is predicted to be 10- to 100-fold higher than disease incidence, suggesting that exposure to *S*. Tm alone is not sufficient to trigger illness (Simonsen et al. 2009). This raises the question which factors determine asymptomatic carriage versus disease, characterised by clinical manifestations such as fever. This disparity is likely at least partially explained by the multi-layered protection provided by the microbiota, the epithelial barrier and the immune system. Here, we will review how these different layers contribute to protection against *S*. Tm infection and address how inter-individual differences in these layers might increase susceptibility to disease. Additionally, we will discuss how *S*. Tm has evolved to overcome this multi-layered protection. This will shed light on the complex interactions between *S*. Tm and the host that determine the outcome of infection.

## The intestinal microbiota: colonisation resistance against *S*. Tm

The intestinal microbiota, comprising bacteria, fungi, viruses, and archaea, is a key component of the multi-layered defences protecting the host against infection. Here, we will specifically focus on the bacterial members of the microbiota, which play an important role in shaping the gut environment through metabolic activity and immune modulation. These mechanisms influence whether exposure to *S*. Tm leads to asymptomatic carriage or symptomatic disease.

The bacterial community varies largely throughout the GI tract, with the greatest microbial density of approximately 10^11^ bacteria per gram content found in the large intestine, predominantly comprising bacteria from the *Firmicutes* and *Bacteroidetes* phyla (Sender et al. 2016; Shalon et al. 2023). In contrast, the small intestine harbours a lower microbial density of approximately 10^8^ bacteria per gram content and has a distinct composition, often including more *Proteobacteria* and *Actinobacteria* (Sender et al. 2016; Shalon et al. 2023). Nutrient availability is an important factor that influences microbiota composition and function. The large intestinal community is mostly fed by complex carbohydrates, including dietary fibres, that cannot be broken down by host enzymes (Zeng et al. 2022). These complex carbohydrates are broken down by the microbiota through carbohydrate-active enzymes, such as glycoside hydrolases and polysaccharide lyases, yielding less complex sugars, including pentoses and hexoses (Wardman et al. 2022). Furthermore, proteins that escape small intestinal enzymatic digestion and therefore reach the large intestine, can be hydrolysed into amino acids and peptides by the microbiota using proteases and peptidases (Diether and Willing 2019). In addition to these diet-derived substrates, host-derived components such as mucus and shed epithelial cells provide glycoproteins and other macromolecules that can be metabolised by microbiota members (Anderson et al. 2021). In the healthy gut, the microbiota ferments these sugars, peptides and amino acids into short-chain fatty acids (SCFAs), acetate, propionate and butyrate, as well as succinate, lactate, formate, carbon dioxide (CO_2_) and hydrogen (H_2_) (Smith and MacFarlane 1998; Fu et al. 2022). Some of these metabolites, including succinate, lactate and formate, can be taken up and fermented by other bacterial species, fostering metabolic cross-feeding among microbiota species (Culp and Goodman 2023). The availability of specific carbohydrates and peptides creates nutritional niches that promote the colonisation of metabolically distinct microbiota species. Consequently, metabolism of both diet-derived and microbiota-derived metabolites shapes microbiota composition and function in the large intestine (Tramontano et al. 2018; Contijoch et al. 2019; Shalon et al. 2023; Little et al. 2024). Understanding these metabolic processes is essential for elucidating how the microbiota contributes to the protection against *S*. Tm infection.

### Colonisation resistance

**Figure 3.**
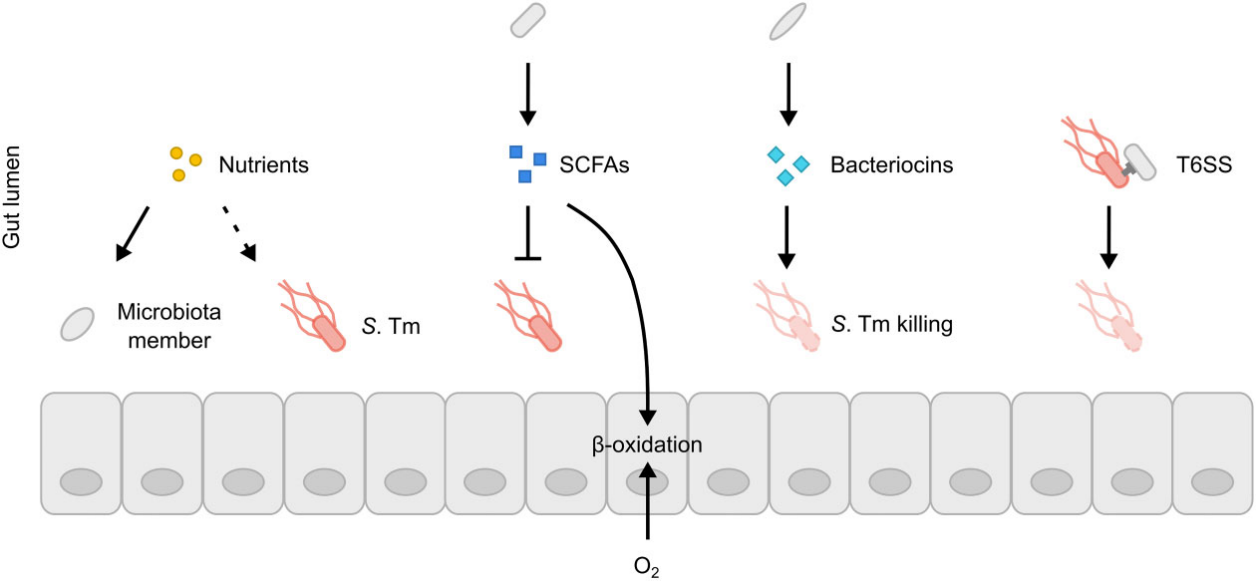
Mechanisms of microbiota-mediated colonisation resistance. The microbiota protects against gut-luminal *S*. Tm colonisation by competing for essential nutrients and producing short-chain fatty acids (SCFAs) that inhibit *S*. Tm growth. Additionally, microbiota members can secrete bacteriocins or express a T6SS that kills *S*. Tm. Together, these mechanisms provide colonisation resistance against *S*. Tm infection.

The microbiota protects against *S*. Tm colonisation through colonisation resistance, primarily by depleting nutrients (exploitation), producing inhibitory factors (interference), and modulating immune responses (Fig. 3). One of the most prominent mechanisms underlying colonisation resistance is nutrient competition. According to Freter's nutrient niche theory, the microbiota effectively occupies a wide range of nutritional niches within the intestinal lumen, which leaves only minimal unoccupied niches for incoming pathogens. By utilising essential nutrients like carbohydrates, amino acids and iron, the microbiota limits their availability to intestinal pathogens (Zeng et al. 2022; Meier et al. 2023). In the murine large intestine, carbohydrate monomer concentrations typically range between 1 millimolar and 1 micromolar (Furuichi et al. 2024; Nguyen et al. 2024; Rogers et al. 2024; Schubert et al. 2025b). These monomers are largely consumed by the microbiota for fermentation but can also fuel *S*. Tm growth by mixed acid fermentation, highlighting the importance of monomer utilisation by the microbiota to limit *S*. Tm colonisation (Nguyen et al. 2024; Rogers et al. 2024; Schubert et al. 2025b). This competition for nutrient niches is particularly effective against closely related bacterial species with similar nutritional needs (Velazquez et al. 2019; Choudhary et al. 2023; Spragge et al. 2023; Laganenka et al. 2025; Lentsch et al. 2025; Schubert et al. 2025a). For example, *Enterobacteriaceae* like *E. coli* and *Klebsiella oxytoca* significantly limit *S*. Tm gut colonisation through competition for galactitol and iron (Deriu et al. 2013; Eberl et al. 2021; Osbelt et al. 2024). Thus, nutrient utilisation by the microbiota is a key contributor to colonisation resistance.

Another mechanism that contributes to colonisation resistance is the production of inhibitory metabolites as well as peptides and proteins. The microbiota produces bacteriocins that act as toxins primarily on closely related species, limiting the ability of these species to colonise the gut lumen (Kommineni et al. 2015; Sassone-Corsi et al. 2016). For example, *E. coli* partially protects against *S*. Tm colonisation of the inflamed gut by producing microcins, like H47, that kill *S*. Tm (Sassone-Corsi et al. 2016; Cherrak et al. 2024, 2025). Furthermore, microbiota-derived SCFAs contribute to colonisation resistance by acidifying the gut lumen, which disrupts pH homeostasis in *S*. Tm and other intestinal pathogens, consequently impairing luminal growth (Jacobson et al. 2018; Sorbara et al. 2019). SCFAs additionally negatively affect T3SS-1 expression by *S*. Tm, reducing its ability to invade host cells and spread systemically (Hung et al. 2013; Hockenberry et al. 2021). Additionally, commensals can use the T6SS to deliver toxins and thereby kill the pathogen and prevent disease (Song et al. 2021; Serapio-Palacios et al. 2022). Together, this emphasises that the microbiota employs multiple mechanisms to protect against gut-luminal *S*. Tm colonisation and thereby reduces the risk of infection of the intestinal tissue.

**Figure 4.**
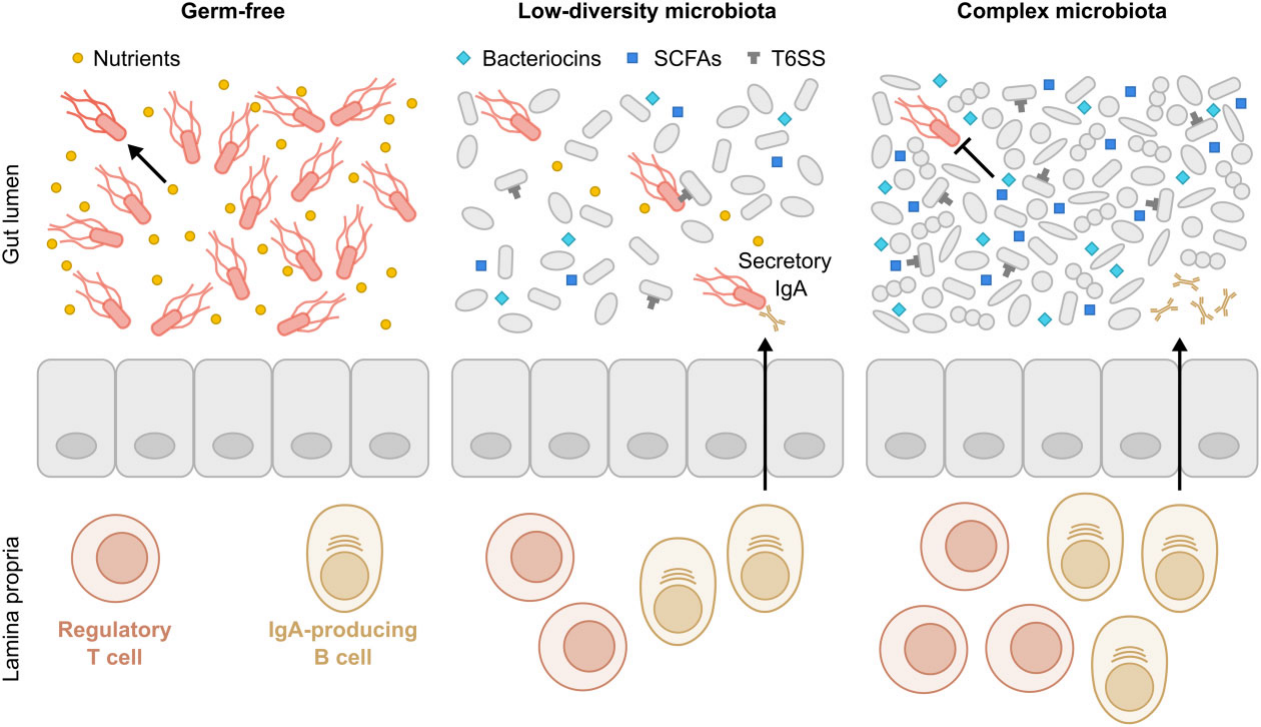
Inverse correlation between microbiota density and *S*. Tm density. In germ-free conditions, where the microbiota is absent, *S*. Tm can exploit the abundance of free nutrients to fuel its colonisation. When a low-diversity microbiota is present, some nutrient niches are occupied, and inhibitory compounds such as short-chain fatty acids (SCFAs) and bacteriocins are produced, leading to partial inhibition of *S*. Tm colonisation. In contrast, a complex microbiota occupies all nutrient niches and spaces, and produces high concentrations of inhibitory compounds, effectively inhibiting *S*. Tm colonisation. Additionally, a complex microbiota drives the maturation of the intestinal immune system and the recruitment and differentiation of regulatory T cells and IgA-producing B cells that contribute to overall intestinal homeostasis and limit *S*. Tm colonisation.

The analysis of pathogen population dynamics using barcoded *S*. Tm consortia and using germ-free and gnotobiotic mouse models have highlighted the microbiota as the primary factor limiting *S*. Tm gut colonisation (Hotinger et al. 2025). In germ-free mice, which lack a microbiota, nutrients are highly abundant, enabling *S*. Tm to reach a high density of 10^9^ colony forming unit (c.f.u.) per gram faeces within 8 h of infection (Fig. 4) (Stecher et al. 2005; Brugiroux et al. 2016; Gül et al. 2023a). Oligo-MM^12^ mice harbour a defined community of 12 microbiota strains that limits nutrient availability and produces bacteriocins and SCFAs, restricting the *S*. Tm density to 10^6^ c.f.u. per gram faeces. In specific pathogen free mice, harbouring a complex microbiota, the competition mechanisms are even stronger, resulting in a low *S*. Tm density of 10^3^ c.f.u. per gram faeces in most animals (Barthel et al. 2003; Brugiroux et al. 2016; Gül et al. 2023a). A recent study further confirmed that the complexity of the microbiota is inversely correlated with gut-luminal *S*. Tm density, as gnotobiotic mice colonised with microbial communities of increasing complexity showed reduced *S*. Tm colonisation in the absence of inflammation (Spragge et al. 2023). Colonisation resistance depended both on the presence of key species such as *E. coli* and interactions among multiple species (Spragge et al. 2023). These findings establish that microbiota complexity is a key component of the host's multi-layered defence against *S*. Tm, underscoring its role in preventing disease.

Disruption of colonisation resistance due to environmental changes, such as drug use or dietary shifts, significantly compromise protection against *S*. Tm infection. Antibiotics, specifically developed to inhibit or kill bacteria, can disrupt the microbiota, which liberates nutrients and reduces inhibitory factor production, opening a niche for *S*. Tm colonisation (Ng et al. 2013; Maier et al. 2021). This has been the basis of the streptomycin mouse model, which is commonly used to study *S*. Tm infection (Barthel et al. 2003). Notably, even non-antibiotic prescription drugs can have off-target effects on the microbiota, affecting its abundance, composition, and metabolism (Maier et al. 2018; Grießhammer et al. 2025; Ricaurte et al. 2024). These drug-induced changes can create a niche that allows for *S*. Tm expansion in the gut lumen (Grießhammer et al. 2025; Ricaurte et al. 2024). Thus, antibiotics represent a double-edged sword in *S*. Tm therapy, eliminating the pathogen and disrupting colonisation resistance. This effect is one of the reasons why antibiotic therapy is generally avoided in cases of self-limiting *S*. Tm diarrhoea. Recent work in mice suggests that therapy schemes permitting the transfer of healthy complex gut microbiota may help to reconstitute colonisation resistance and thereby prevent relapses after the end of the antibiotic therapy (Newson et al. 2025).

Shifts in diet represent another environmental factor that can significantly affect colonisation resistance. For example, a shift to a high-fat diet alters nutrient availability and increases bile production, which impacts microbiota composition and favours *S*. Tm, as it exhibits higher resistance to bile salts than most microbiota members (David et al. 2014; Sonnenburg et al. 2016; Wotzka et al. 2019; Kokkinias et al. 2024). Similarly, a low-fibre diet reduces the availability of fibres, prompting mucus degradation by *Akkermansia muciniphila* and *Bacteroides caccae*, which utilise glycans from the mucus layer as an alternative nutrient source (Desai et al. 2016; Wotzka et al. 2019; Ostrem Loss et al. 2023). This degradation impairs the protection provided by the mucus layer against pathogenic invasion of IECs. Interestingly, while acute fasting also affects gut-luminal nutrient availability, it surprisingly enhances colonisation resistance against *S*. Tm and suppresses virulence of *S*. Tm through mechanisms that are incompletely understood (Graef et al. 2021). This highlights how changes in the gut-luminal environment can either negatively or positively affect colonisation resistance. Importantly, shifts to a high-fat or low-fibre diet can also negatively impact the metabolic fitness of intestinal epithelial and immune cells, compromising the host's overall protection against infection (Wotzka et al. 2019; Yoo et al. 2021; Phiri et al. 2023; Siracusa et al. 2023). Furthermore, such dietary changes can have lasting effects on the metabolic function of individual microbiota members, thereby reducing long-term colonisation resistance against *S*. Tm (Dapa et al. 2022). These findings underscore the role of environmental factors in disrupting colonisation resistance, potentially contributing to the onset of disease in *S*. Tm patients.

### Host-microbiota interactions

The microbiota also plays a role in shaping the host's immune system, particularly during early life (Chung et al. 2012; De Agüero et al. 2016; Lubin et al. 2023). At birth, the immune system and lymphoid organs are immature, and exposure to the microbiota during early life is essential for immune maturation (De Agüero et al. 2016). For example, microbiota exposure drives the formation of mLNs and GALT (Yamanaka et al. 2003; Borbet et al. 2023). Furthermore, the introduction of solid food during weaning and subsequent changes in microbiota composition, increases the abundance of both myeloid and lymphoid cells in the intestine and induce secretory immunoglobulin A (IgA) production (Hapfelmeier et al. 2010; Chung et al. 2012; De Agüero et al. 2016). An essential component of this maturation process is the induction of RORγt^+^ regulatory T cells (Tregs) in the lamina propria, which establish immune tolerance to the microbiota and prevent the onset of chronic intestinal inflammation (Ohnmacht et al. 2015; Sefik et al. 2015; Al Nabhani et al. 2019). Notably, disruptions in the microbiota during early life are associated with abnormal immune system development and increased susceptibility to *S*. Tm infection, including elevated gut-luminal colonisation and systemic dissemination (Lubin et al. 2023). Thus, early host-microbiota interactions imprint an anti-inflammatory state in the intestinal immune system, which establishes homeostasis of the multi-layered intestinal barrier and provides protection against infection, thereby influencing the host's susceptibility to *S*. Tm-mediated disease.

This influence of the microbiota on the host extends into adulthood, where it continues to shape host physiology, particularly through the production of SCFAs. SCFAs are absorbed by IECs via passive diffusion or active transport mechanisms (Ruppin et al. 1980; Kim 2021). Butyrate, in particular, serves as an important energy source for IECs, enhancing epithelial barrier function (Roediger 1982; Donohoe et al. 2011; Kelly et al. 2015). Notably, butyrate metabolism by IECs consumes oxygen and thereby maintains anaerobiosis in the gut lumen (Rivera-Chávez et al. 2016b). SCFAs that are not metabolised by IECs enter the lamina propria and the systemic circulation, where they can act on G-protein-coupled receptors (GPCRs) expressed by host cells, including immune cells (Maslowski et al. 2009; Chen et al. 2019). This GPCR-signalling promotes an anti-inflammatory phenotype in intestinal myeloid cells and T cells, further supporting the anti-inflammatory status of the intestine (Smith et al. 2013; Singh et al. 2014). Together, the production of SCFAs by the microbiota aids intestinal homeostasis throughout life. Reductions in gut-luminal SCFA concentrations not only indicate decreased microbiota fermentation and reduced acidification of the gut lumen, but can also negatively affect the host, potentially altering IEC metabolism and influencing the inflammatory state of immune cells. This indicates that SCFA production by the microbiota represents an important factor contributing to protection against symptomatic disease.

The interactions between the host and microbiota during early life and adulthood establish a symbiotic relationship that is essential for maintaining overall intestinal health and protection against infections. This interplay is bidirectional; the host also influences the microbiota. Conversely, disturbances in the host's anti-inflammatory status, triggered by pro-inflammatory responses to local intestinal or systemic stimuli, have an impact on the microbiota and thus on the protection against *S*. Tm-mediated disease (Becattini et al. 2021; Stacy et al. 2021; Tawk et al. 2023; Kroon et al. 2025). For example, local intestinal inflammation caused by *S*. Tm infection disrupts colonisation resistance, increasing susceptibility to intestinal infection (Stecher et al. 2007; Rivera-Chávez et al. 2016b). However, in the long term, infection-induced intestinal inflammation enriches the microbiota for members that are more resistant to inflammatory conditions, thereby enhancing colonisation resistance to subsequent infections (Stacy et al. 2021; Tawk et al. 2023). In contrast, systemic immune activation does not directly alter microbiota composition but instead induces transient changes in microbiota metabolism (Becattini et al. 2021). Strikingly, systemic immune activation increases gut-luminal *S*. Tm colonisation through elevated gut-luminal oxygen species levels, which inhibits the microbiota and enables aerobic respiration by *S*. Tm (Kroon et al. 2025). Additionally, systemic immune activation triggers bacterial translocation from the gut lumen to the liver by reducing the production of antimicrobial peptides (AMP) by Paneth cells (Wallaeys et al. 2024). We hypothesise that the transient increase in oxygen species and reduced AMP secretion observed during systemic immune activation might also be triggered locally during *S*. Tm infection of gut tissue. These localised responses could transiently alleviate colonisation resistance near the site of infection, facilitating gut luminal pathogen growth and systemic dissemination (Hausmann et al. 2021; Liou et al. 2022). These observations highlight that local and systemic pro-inflammatory responses impact colonisation resistance and bacterial translocation, potentially contributing to *S*. Tm-mediated disease.

Taken together, the microbiota contributes to the multi-layered protection against *S*. Tm infection by providing colonisation resistance. Both the composition and functional capacity of the microbiota influence the strength of colonisation resistance through mechanisms such as nutrient competition, production of inhibitory compounds, and immune modulation (Ruddle et al. 2023; Yoo et al. 2024; Kroon et al. 2025). The presence of closely related microbiota strains, particularly those within the *Enterobacteriaceae* family, is especially effective at inhibiting *S*. Tm colonisation by competing for similar niches and resources, thereby reducing the risk of *S*. Tm-mediated disease (Velazquez et al. 2019). Perturbations in the gut environment driven by factors, such as drug use, dietary shifts, and pro-inflammatory responses, can disrupt microbiota homeostasis and thereby increase susceptibility to *S*. Tm-mediated disease (Wotzka et al. 2019; Maier et al. 2021; Grießhammer et al. 2025). These factors alter nutrient availability, shift microbiota composition, or disrupt the protective mucus barrier, creating niches that favour *S*. Tm expansion (Wotzka et al. 2019; Maier et al. 2021; Grießhammer et al. 2025). This change in the environment can additionally increase virulence expression by *S*. Tm, further increasing the likelihood of *S*. Tm to reach and invade the epithelial barrier and thereby enhance its potential to trigger disease. Thus, the interplay between microbiota composition, function, and environmental factors plays an important role determining whether *S*. Tm exposure results in asymptomatic carriage or symptomatic disease.

## Intestinal epithelial barrier: a physical barrier against *S*. Tm

The intestinal epithelial barrier consists of a single layer of IECs, which are important for nutrient absorption and serve as a key component of the multi-layered protection against intestinal *S*. Tm infection (Hausmann et al. 2024). In the small intestine, this barrier is characterised by a structure of crypts and villi, while in the large intestine, it appears smooth but retains crypts. Within these crypts, leucine-rich repeat-containing G protein-coupled receptor 5 (LGR5^+^) stem cells give rise to IEC progenitors (Barker et al. 2007; Sato et al. 2009; Rodríguez-Colman et al. 2017). Stem cell proliferation is driven by wingless-integrated (Wnt) signalling induced by mesenchymal cells, macrophages and Paneth cells (Sato et al. 2011; Degirmenci et al. 2018; Kim et al. 2022). As progenitor cells move into the transit-amplifying zone, they continuously proliferate and commit to the absorptive or secretory cell lineage in response to Notch and interleukin-17 (IL-17) signalling (Van Es et al. 2005; Krndija et al. 2019; Lin et al. 2022). Further along the crypts and villi, bone morphogenetic protein (BMP) produced by mesenchymal cells drives terminal differentiation of IECs (He et al. 2004; McCarthy et al. 2020; Kraiczy et al. 2023). These tightly regulated processes, involving multiple signalling pathways and cellular interactions ensure the continuous renewal and function of the intestinal epithelial barrier, which is a critical component of the multi-layered protection against *S*. Tm infection. Additionally, these IEC turnover mechanisms enable rapid restoration of the barrier following *S*. Tm infection, limiting pathogen translocation and reducing the risk of disease progression.

**Figure 5.**
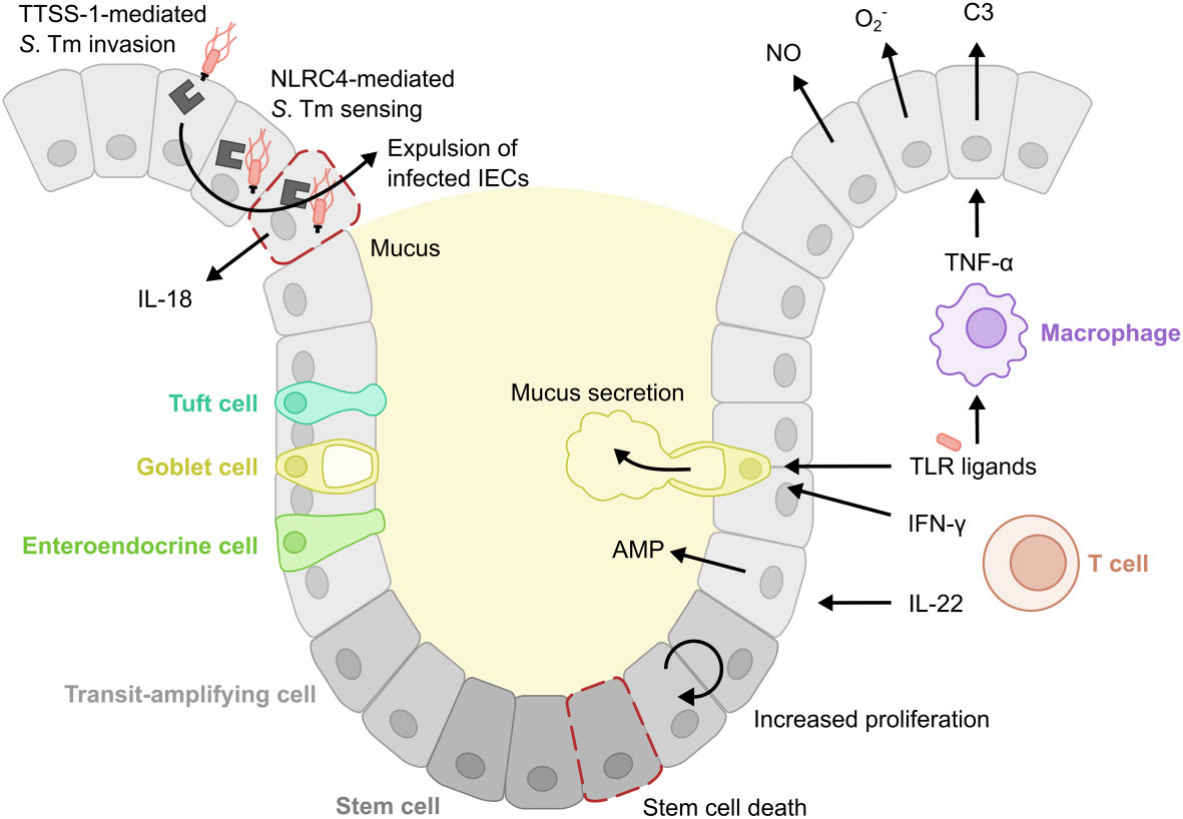
Epithelial responses to *S*. Tm infection of the mouse caecum. Upon infection by *S*. Tm, intestinal epithelial cells (IECs) activate the NAIP/NLRC4 inflammasome, triggering the expulsion of infected cells to clear the epithelial barrier. This response results in stem cell death and stimulates the proliferation of transit-amplifying cells to restore barrier integrity. Simultaneously, cytokine production in the lamina propria in response to *S*. Tm detection enhances mucus secretion, antimicrobial peptide (AMP) release, and the production of reactive species, including superoxide (O_2_^−^) and nitric oxide (NO), collectively limiting the ability of *S*. Tm to reach and invade the intestinal barrier.

The microbiota shapes a specific intestinal program in IECs that is characterised by high tolerance to pathogen-associated molecular patterns (PAMPs), such as lipopolysaccharide (LPS) (Lotz et al. 2006; Price et al. 2018). PAMP tolerance is established early in the neonatal period through negative regulation of Toll-like receptors (TLRs) signalling pathways (Chassin et al. 2010; Sham et al. 2013). Interestingly, some microbiota members, such as *Lachnospiraceae* spp., produce flagellins that are weak TLR5 agonists, and therefore do not trigger the same type of responses as flagellin from pathogens (Clasen et al. 2023). This might represent mechanisms by which both the host and microbiota have adapted to maintain symbiosis. Upon infection, antimicrobial IEC responses are primarily triggered by cytokines, rather than through TLR signalling in IECs (Kinnebrew et al. 2012; Price et al. 2018; Hausmann et al. 2021). For example, TNF-α triggers the release of complement 3 (C3) by IECs which can protect against intestinal infection, and IL-22 stimulates AMP Reg-IIIγ secretion (Kinnebrew et al. 2012; Hausmann et al. 2021; Wu et al. 2024) (Fig. 5). Thus, IECs activate distinct responses depending on the type of signal, enabling them to specifically target pathogenic threats (Kinnebrew et al. 2012; Price et al. 2018; Hausmann et al. 2021). This immunoregulatory network prevents intestinal inflammation in response to abundant gut-luminal PAMPs, maintaining homeostasis within the multi-layered intestinal barrier, while also driving potent antimicrobial responses during infection.

IECs also play an active role in shaping the microbiota composition and maintaining host-microbiota symbiosis. Under homeostasis, the microbiota drives the expression of NADPH oxidases and inducible nitric oxide synthase (iNOS) by IECs, leading to low-level production of reactive oxygen species (ROS) and reactive nitrogen species (RNS) (Grasberger et al. 2015; van der Post et al. 2021). In addition, the microbiota induces the production of low levels of AMPs by IECs (Cash et al. 2006; Vaishnava et al. 2011; Grasberger et al. 2015; Matziouridou et al. 2018; Yardeni et al. 2019). This controlled production of ROS, RNS and AMPs shapes the microbiota composition and ensures that, in the colon, the microbiota only colonises the outer mucus layer and not the inner mucus layer, enhancing the physical separation from the epithelium (Vaishnava et al. 2011; Grasberger et al. 2015; Bergström et al. 2016; Matziouridou et al. 2018; Yardeni et al. 2019). These mechanisms underscore the crucial role of IEC responses in maintaining host-microbiota homeostasis and protecting against systemic dissemination of the microbiota. Together, this illustrates the evolutionary adaptations of both the host and microbiota to sustain symbiosis, which is essential for epithelial integrity and protection against *S*. Tm infection.

### Role of epithelial cells in protection against *S*. Tm infection

The intestine comprises a multitude of cell types with distinct localisations and specialised functions. This includes absorptive enterocytes and secretory IECs, like enteroendocrine cells, goblet cells, tuft cells and Paneth cells (Fig. 5). Together, these cells contribute to the multi-layered protection against *S*. Tm infection.

The majority of IECs are enterocytes, which are essential for nutrient absorption. Enterocytes exhibit distinct functional capabilities based on their location along the crypt-villus axis to ensure overall intestinal health (Moor et al. 2018). Specifically, enterocytes in close proximity to the stem cell crypt exhibit an antimicrobial transcriptional program, mid-villus enterocytes perform nutrient absorption, and those at the villus tip have immune-modulatory functions (Moor et al. 2018). Strikingly, *S*. Tm infection is characterised by both stem cell death and an increase in cell proliferation in the transit-amplifying zone (Hapfelmeier et al. 2005; Haber et al. 2017; Fattinger et al. 2021; Yan et al. 2024). These epithelial responses are driven by the activation of the inflammasome by *S*. Tm virulence factors, as described below. This response limits the number of infected IECs which remain in the mucosa, accelerates intestinal inflammation, and alters the signalling pathways that regulate the differentiation of progenitor cells (Sellin et al. 2014; Rauch et al. 2017; Fattinger et al. 2021, 2023). During *S*. Tm infection, transit-amplifying cells primarily differentiate into absorptive enterocytes rather than the secretory cells, which may take longer to fully differentiate (Yan et al. 2024). This may effectively preserve barrier integrity and maintain essential nutrient absorption at the expense of full differentiation. This underscores the importance of enterocyte plasticity in preserving protection against *S*. Tm infection.

Goblet cells, highly abundant in the large intestine, produce granules packed with mucin-2 (Muc2), which can be secreted into the intestinal lumen to form a mucus layer that acts as a physical barrier between the IECs and the microbiota (Furter et al. 2019; Gustafsson and Johansson 2022). The large intestinal mucus layer consists of two layers, an inner mucus layer that is densely packed with Muc2, devoid of bacteria, and an outer mucus layer colonised by the microbiota (Johansson et al. 2008; Bergstrom et al. 2020). This mucus layer is produced by two types of goblet cells: crypt-resident goblet cells, which produce mucus within the crypts, and intercrypt goblet cells, which fill the spaces between crypts (Nyström et al. 2021). In the small intestine and caecum, goblet cells are less abundant, resulting in a more porous mucus layer that primarily covers the crypts and leaves their tips exposed, thereby increasing the accessibility of *S*. Tm to IECs (Furter et al. 2019). Upon *S*. Tm infection, interferon-γ (IFN-γ) and TLR signals enhance mucus production by goblet cells to limit access of *S*. Tm to IECs (Songhet et al. 2011; Birchenough et al. 2016). This is further promoted by mucus production by a sentinel goblet cell located in large intestinal crypts, which triggers adjacent goblet cells to also release mucus (Birchenough et al. 2016). The role of the mucus layer in the protection against *S*. Tm infection is underscored by the observation that *Muc2*^−/−^ mice develop spontaneous colitis in response to a normal gut microbiota (Van der Sluis et al. 2006; Zarepour et al. 2013). In *Muc2*^−/−^ mice, *S*. Tm infection leads to exacerbated tissue-disruptive intestinal inflammation and increased susceptibility to infection compared to control mice (Van der Sluis et al. 2006; Zarepour et al. 2013). This highlights the importance of goblet cell-produced mucus in the multi-layered protection against *S*. Tm infection.

Paneth cells, located in small intestinal crypts, produce granules containing AMPs, such as defensins and lysozyme, which also contribute to the multi-layered protection against *S*. Tm infection (Vaishnava et al. 2008; Kamioka et al. 2022). These AMPs maintain segregation between the microbiota and IECs (Vaishnava et al. 2008; Kamioka et al. 2022). This function is particularly important in the small intestine, where the porous mucus layer does not limit bacterial access to the epithelium (Atuma et al. 2001). Upon infection, IFN-γ triggers rapid Paneth cell degranulation and cell extrusion, releasing AMPs into the gut lumen (Farin et al. 2014; Bel et al. 2017). However, *S*. Tm can interfere with Paneth cell granule formation to evade this defence mechanism (Farin et al. 2014; Bel et al. 2017). In response, Paneth cells employ secreted autophagy to release lysozyme, thereby reducing small intestinal luminal *S*. Tm colonisation and systemic dissemination by 10-fold (Bel et al. 2017). This underscores the role of Paneth cells in sustaining defence mechanisms against *S*. Tm infection.

Tuft cells and enteroendocrine cells are chemo-sensory cells primarily located between mature enterocytes in the small and large intestine (Schneider et al. 2019; Arora et al. 2021). Tuft cells play a crucial role in raising Th2 immunity to protect against helminth infection (Gerbe et al. 2016). Interestingly, recent work has demonstrated that sensing of microbiota-derived butyrate inhibits tuft cell differentiation, whereas sensing of metabolites produced by enteric pathogen *Shigella* triggers AMP production (Xiong et al. 2022b; Eshleman et al. 2024). This indicates that tuft cells employ distinct mechanisms to maintain microbiota homeostasis and respond to pathogenic infection. Enteroendocrine cells produce vesicles that contain hormones, such as glucagon-like peptide-1 (GLP-1), peptide YY and serotonin, which regulate nutrient absorption, insulin secretion, appetite and gut motility. Upon sensing of parasite-induced IL-33 or bacterial-derived tryptophan, enteroendocrine cells secrete serotonin to increase gut motility, driving the expulsion of pathogens (Chen et al. 2021b; Ye et al. 2021). Furthermore, enteroendocrine cell-derived serotonin negatively regulates virulence factor expression by *Citrobacter rodentium* (Kumar et al. 2020). Together, this suggests that enteroendocrine cells and tuft cells respond to pathogenic infection, but their specific contribution to the multi-layered defence mechanisms against *S*. Tm infection requires further investigation.

### Mechanism of epithelial cell death in response to *S*. Tm infection

When the microbiota, mucus layer and AMP production fail to prevent *S*. Tm infection of IECs, IECs employ pyroptosis, a form of pro-inflammatory cell death, to expel infected cells into the gut lumen (Knodler et al. 2014; Sellin et al. 2014; Rauch et al. 2017). Pyroptosis is initiated by nucleotide-binding oligomerisation domain (NOD)-like receptors (NLRs) that assemble inflammasomes upon pathogen recognition (Pandey et al. 2021). Key NLRs include NLR family pyrin domain containing 3 (NLRP3), NLR family CARD domain-containing protein 4 (NLRC4) and NLR family apoptosis inhibitory proteins (NAIPs), with the NAIP/NLRC4 inflammasome being particularly effective against *S*. Tm infection (Franchi et al. 2012; Sellin et al. 2014). NAIPs recognise distinct structural and virulence components of pathogens, such as the T3SS needle protein, T3SS rod protein, or flagellin of *S*. Tm (Kofoed and Vance 2011; Zhao et al. 2011). Upon recognition, NAIPs associate with inactive NLRC4 to assemble the NAIP/NLRC4 inflammasome (Tenthorey et al. 2017; Matico et al. 2024). The NAIP/NLRC4 inflammasome recruits ASC and pro-caspase-1, resulting in the activation of caspase-1, which then cleaves pro-IL-1β, pro-IL-18 and inactive gasdermin D (GSDMD) (Franchi et al. 2006; Lara-Tejero et al. 2006; Miao et al. 2006, 2010). GSDMD forms pores in the cell membrane, triggering pyroptosis and releasing active IL-1β and IL-18, which promote pro-inflammatory responses (Miao et al. 2010; Liu et al. 2016). This cell death mechanism is effectively activated in IECs, as *S*. Tm expresses high levels of flagella and T3SS-1 during initial invasion (Hausmann et al. 2020). The significance of NLCR4/NAIP has been demonstrated in *Nlrc4*^−/−^ mice, which show 50-fold higher *S*. Tm loads in the caecum tissue compared to *Nlrc4*^+/−^ littermate controls at 18 h post infection (Fig. 5) (Sellin et al. 2014). In these *Nlrc4*^−/−^ mice, which cannot control epithelial *S*. Tm loads, increased lamina propria TNF-α levels drive barrier dislodgement by day 3 post infection, indicating that the NAIP/NLRC4 inflammasome is also required to prevent late-stage barrier disruption (Fattinger et al. 2021). Furthermore, this mechanism not only prevents *S*. Tm invasion into the lamina propria but also inhibits the ability of *S*. Tm to utilise cytosolic IEC replication to fuel faecal shedding (Chong et al. 2021). This highlights how pyroptosis of infected IECs serves as another layer of protection during *S*. Tm infection.

Recent studies have more specifically focussed on the role of pyroptosis effector GSDMD in the protection of IECs against *S*. Tm infection (Xiong et al. 2022a; Fattinger et al. 2023). Similar to the NAIP/NLRC4 inflammasome, GSDMD is required for the protection against infection of IECs at 18 h post infection (Fattinger et al. 2023). Interestingly, GSDMD not only localises to host cell membranes but also forms pores in mitochondrial inner and outer membranes, neutrophil granules, and even bacterial membranes (Liu et al. 2016; Karmakar et al. 2020; Weindel et al. 2022; Miao et al. 2023). This pore-forming activity could be particularly significant in IECs, where GSDMD-mediated pore formation might directly target *S*. Tm or disrupt the SCV. The exact mechanism by which GSDMD provides protection against *S*. Tm in IECs requires further investigation.

Collectively, the epithelial barrier plays a central role in the multi-layered protection against *S*. Tm infection by acting as a physical and immunological barrier. Key mechanisms that maintain barrier integrity include continuous epithelial cell turnover, mucus secretion, AMP production, and inflammasome-mediated expulsion of infected IECs. Whether *S*. Tm exposure results in asymptomatic carriage or symptomatic illness partially depends on changes in the integrity and functionality of the intestinal barrier. Disruptions in barrier integrity, often driven by comorbidities like inflammatory bowel disease (IBD) and diabetes, increase the probability of *S*. Tm translocation into the sterile compartment and thus affect the susceptibility to infection (Swidsinski et al. 2007; Irving et al. 2021). Additionally, genetic polymorphisms and shifts in microbiota composition alter antimicrobial defence mechanisms of IECs, which may impair effective protection against *S*. Tm infection. Developmental differences further shape infection outcomes. Infant mice lack inflammasome-dependent expulsion of IECs, suggesting that NAIP/NLRC4-mediated pyroptosis may not be fully functional in neonatal hosts (Zhang et al. 2024). Thus, the outcome of *S*. Tm exposure is influenced by a combination of the developmental stage, underlying disease states, genetic predisposition, and microbiota composition, indicating that multiple factors determine the ability of the epithelial barrier to protect against *S*. Tm-mediated disease.

## The intestinal immune system: antimicrobial responses to *S*. Tm

The final layer of protection is provided by the intestinal immune system. As the intestinal immune system is equipped with robust antimicrobial response mechanisms, it continuously balances tolerance to the microbiota while restricting pathogenic invasion. The microbiota drives the maturation of the intestinal immune system and imprints an anti-inflammatory and tolerogenic state in intestinal immune cells, characterised by the production of IL-10 and transforming growth factor β (TGF-β) (Zigmond et al. 2014; De Agüero et al. 2016). However, during *S*. Tm infection this balance shifts, as the innate immune system, including neutrophils, monocytes, macrophages and cDCs, orchestrates a robust pro-inflammatory response. This response is primarily induced via TLRs and NLRs, and is characterised by the production of cytokines, such as IL-1β, TNF-α and IFN-γ, which drive antimicrobial programs for pathogen clearance and the induction of adaptive immune responses (Liu et al. 2010; Dolowschiak et al. 2016; Xiong et al. 2019). Thus, the immune system plays key roles in both intestinal homeostasis and protection against infection. Here, we will explore how various immune cells contribute to these protective functions and thereby limit the risk of symptomatic disease progression.

### Intestinal neutrophils

Neutrophils are potent antimicrobial cells equipped with intracellular granules containing elastases, proteases and myeloperoxidases (Burn et al. 2021). They produce ROS, AMPs and neutrophil extracellular traps (NETs) in response to bacterial sensing. This antimicrobial program is innately imprinted into neutrophil progenitors in the bone marrow and develops further during neutrophil maturation (Clarke et al. 2010; Zhang et al. 2015; Grassi et al. 2018; Xie et al. 2020). Under homeostasis, neutrophil abundance in the intestinal tissue is limited, with those present preferentially locating in ILFs rich in B cells and CD169^+^ macrophages, where they play a role in angiogenesis (Casanova–Acebes et al. 2018; Ballesteros et al. 2020). Interaction of neutrophils with macrophages in ILFs downregulates granulocyte-colony stimulating factor (G-CSF) production by macrophages, limiting the release of neutrophil progenitor cells into the bloodstream (Casanova–Acebes et al. 2018). This mechanism may dampen neutrophil recruitment into the intestinal tissue during homeostasis, preserving the integrity of the multi-layered intestinal barrier by limiting the infiltration of antimicrobial neutrophils.

**Figure 6.**
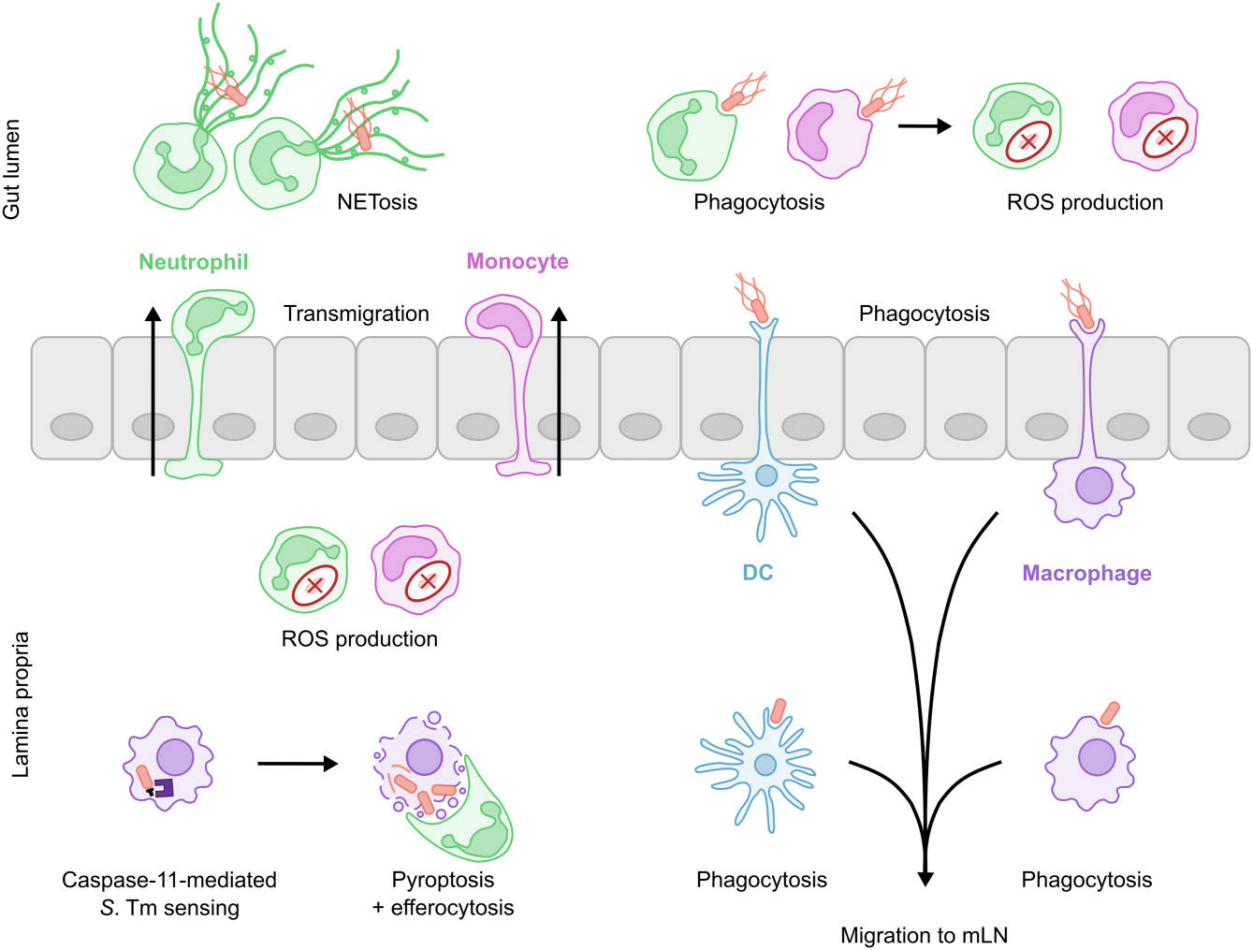
Phagocyte responses to *S*. Tm infection. Phagocytes employ a range of mechanisms to restrict *S*. Tm infection. Neutrophils effectively kill *S*. Tm in the lamina propria through reactive oxygen species (ROS) production. They can also transmigrate into the gut lumen, where they utilise both ROS and NETosis to target gut-luminal *S*. Tm, reducing epithelial invasion. Monocytes similarly employ ROS-mediated killing in both the lamina propria and the gut lumen. Macrophages and dendritic cells (DCs) extend dendrites through the epithelium to sample the gut lumen and phagocytose *S*. Tm in the lamina propria. These cells then migrate to the mesenteric lymph nodes (mLN) to initiate adaptive immune responses. To restrict *S*. Tm replication within macrophages, macrophages trigger caspase-11-mediated pyroptosis, which traps *S*. Tm in cell debris and rendering it vulnerable to neutrophil efferocytosis and killing.

During *S*. Tm infection, neutrophils act as first responders. Binding of *S*. Tm to IECs triggers the release of CXCL8, rapidly recruiting large numbers of neutrophils into the intestinal tissue (McCormick et al. 1995). Neutrophils then transmigrate into the intestinal lumen, where they eliminate a significant portion of luminal *S*. Tm (Fig. 6) (Mrsny et al. 2004; Maier et al. 2014; Gül et al. 2023b). The impact of this has been demonstrated in mice treated with an anti-Gr1 antibody, depleting neutrophils and monocytes, which showed 10-fold higher gut-luminal *S*. Tm loads at 2 days post infection compared to isotype control treated mice (Maier et al. 2014). Furthermore, neutrophils release DNA, cytosolic proteins, granule proteins and AMPs to form NETs, which trap and kill *S*. Tm and thereby prevent access to the epithelium (Molloy et al. 2013; Branzk et al. 2014; Gül et al. 2023b). Together, these mechanisms provide protection against *S*. Tm invasion of the epithelial barrier.

When *S*. Tm reaches the intestinal lamina propria, neutrophils migrate towards inflammatory signals to locate and target *S*. Tm (Kienle et al. 2021). Upon *S*. Tm uptake, neutrophils employ NADPH oxidases for ROS-mediated killing of *S*. Tm in the phagosome (Burton et al. 2014). To protect the host against ROS that diffuses out of the neutrophil phagosome into the tissue, neutrophils utilise myeloperoxidases to convert highly diffusible hydrogen peroxide (H_2_O_2_) into less diffusible hypochlorous acid (HOCl) (Schürmann et al. 2017). Hypochlorous acid directly acts on the *S*. Tm cell surface, efficiently killing it (Schürmann et al. 2017). Simultaneously, granule proteases cleave IL-1 family cytokines, including IL-1α and IL-1β, thereby amplifying the pro-inflammatory response (Clancy et al. 2018). Thus, this multi-faceted neutrophil approach not only kills *S*. Tm but also enhances the overall immune response.

Despite the antimicrobial properties of neutrophils, *S*. Tm can occasionally survive within them, presumably by employing virulence factors for replication. However, T3SS-1-mediated invasion of neutrophils by *S*. Tm triggers inflammasome activation, and subsequent cleavage of pro-GSDMD (Chen et al. 2014, 2018; Karmakar et al. 2020). Unlike in IECs or macrophages, where inflammasome activation triggers pyroptosis, in neutrophils, GSDMD localises to granules, resulting in degranulation (Karmakar et al. 2020). Alternatively, the recognition of cytosolic LPS drives caspase-11-mediated GSDMD cleavage, leading to NETosis (Chen et al. 2018). This indicates that recognition of T3SSs and LPS trigger distinct neutrophil responses, both aimed at *S*. Tm clearance though GSDMD-mediated mechanisms.

Taken together, neutrophils play a crucial role in the multi-layered protection against *S*. Tm. This is particularly evident in patients with chronic granulomatous disease, where the absence of functional NADPH oxidase in neutrophils impairs their ability to produce ROS and effectively kill *S*. Tm (van den Berg et al. 2009). As a result, *S*. Tm is the leading cause of gastroenteritis and bloodstream infections in these patients (van den Berg et al. 2009). Neutrophil recruitment and function are thus important contributors to the protection against *S*. Tm-mediated disease.

### Intestinal monocytes and macrophages

Murine intestinal macrophages reside in the lamina propria, where they exhibit a non-migratory, anti-inflammatory and highly phagocytic phenotype (Viola and Boeckxstaens 2021). In contrast to many tissue-resident macrophages that derive from embryonic yolk sac or foetal liver precursors, the majority of intestinal macrophages are short-lived and are constantly replenished by blood monocytes derived from the bone marrow (Yona et al. 2013; Bain et al. 2014; Khosravi et al. 2014). The microbiota drives the recruitment of Ly-6C^hi^ CX3CR1^low^ monocytes into the intestinal tissue, where TGF-β, IL-10 and GM-CSF promote their maturation into anti-inflammatory CX3CR1^hi^ macrophages (Tamoutounour et al. 2012; Bain et al. 2013, 2014; Zigmond et al. 2014; Schridde et al. 2017; Kvedaraite et al. 2024). Microbiota-derived butyrate metabolically reprograms intestinal macrophages towards oxidative phosphorylation (OXPHOS), reinforcing their anti-inflammatory state (Scott et al. 2018; Schulthess et al. 2019). Consequently, these CX3CR1^hi^ macrophages exhibit tolerance to PAMPs and produce anti-inflammatory cytokines, such as IL-10 (Smythies et al. 2005; Denning et al. 2007). These macrophages are non-motile and remain in the lamina propria, preventing the induction of adaptive immune responses in the mLNs (Diehl et al. 2013; Kim et al. 2018). Locally, they inhibit pro-inflammatory T helper 1 (Th1) cells, while promoting Tregs and T helper 17 (Th17) cells, thus contributing to oral tolerance (Hadis et al. 2011; Diehl et al. 2013; Panea et al. 2015; Kim et al. 2018). Importantly, dysregulation of the anti-inflammatory capacity of intestinal macrophages drives inflammatory intestinal pathogenesis (Bernshtein et al. 2019; Corbin et al. 2020). Thus, the intestinal microenvironment imprints a specific anti-inflammatory phenotype in intestinal macrophages that contributes to homeostasis of the multi-layered intestinal barrier.

Intestinal macrophages vary phenotypically based on their spatial localisation (Kang et al. 2020). CD11c^+^ macrophages, located close to the epithelium, are involved in immune effector functions, aiding the maintenance of the epithelial stem cell niche and IEC turnover, thereby supporting barrier integrity (Sehgal et al. 2018; Chikina et al. 2020; Fritsch et al. 2023). Their location enables them to clear apoptotic IECs and infiltrating pathogens, and sample for luminal antigens (Müller et al. 2012; Morita et al. 2019). In contrast, CD169^+^ macrophages, located closer to the vasculature, contribute to cell recruitment, scavenging and tissue regeneration (Asano et al. 2015; Kang et al. 2020). Recent studies have identified a subset of Tim-4 expressing intestinal macrophages, which are long-lived and may be locally maintained or replenished at a slow rate (Shaw et al. 2018). The ontogeny of these Tim-4^+^ macrophage populations remains to be elucidated, but they are associated with maintaining neuronal and vascular networks (De Schepper et al. 2018; Viola et al. 2023). This underscores the multifaceted role of macrophages in the multi-layered intestinal barrier.

During *S*. Tm infection, there is a shift from a homeostatic anti-inflammatory state to a pro-inflammatory and antimicrobial monocyte/macrophage phenotype. *S*. Tm infection triggers the recruitment of immature CX3CR1^low^ monocytes into the intestinal tissue, which partially transmigrate into the gut lumen (Hapfelmeier et al. 2008; Man et al. 2017). Unlike during homeostasis, these CX3CR1^low^ monocytes do not mature into anti-inflammatory CX3CR1^hi^ macrophages. Instead, CX3CR1^low^ monocytes act as potent pro-inflammatory mediators that employ ROS production for *S*. Tm killing, at which they are more effective than CX3CR1^hi^ macrophages (Burton et al. 2014). This suggests that the pro-inflammatory environment drives monocytes to a state optimised for pathogen clearance. In contrast, CX3CR1^hi^ macrophages primarily contribute to initiating adaptive immune responses against *S*. Tm. They secrete chemokines that recruit T and B cells, initiating a *S*. Tm-specific IgA response (Diehl et al. 2013; Koscsó et al. 2020). Furthermore, upon *S*. Tm infection, CX3CR1^hi^ macrophages acquire CCR7, enabling them to migrate to the mLN to further induce T and B cell responses (Diehl et al. 2013). This underscores the distinct roles that monocytes and macrophages play in the multi-layered protection against *S*. Tm infection: monocytes are more effective at mediating *S*. Tm killing, while macrophages are crucial for orchestrating adaptive immunity.

Importantly, *S*. Tm can exploit CX3CR1^hi^ macrophages for replication within the lamina propria. TLR activation of these cells drives phagosome acidification, facilitating the formation of the protective SCV that supports *S*. Tm replication (Arpaia et al. 2011). In the lamina propria *S*. Tm downregulates the expression of T3SS-1 and flagellin, resulting in limited activation of NAIP/NLRC4-mediated pyroptosis in CX3CR1^hi^ macrophages (Hausmann et al. 2020). As an alternative mechanism, CX3CR1^hi^ macrophages activate IFN-inducible guanylate-binding proteins (GBPs) that can lyse the SCV, releasing LPS or *S*. Tm into the cytosol (Meunier et al. 2014; Pilla et al. 2014). Caspase-11 then senses cytoplasmic LPS and cleaves inactive GSDMD, triggering pyroptosis (Hagar et al. 2013; Kayagaki et al. 2013; Shi et al. 2014). Although this induction of cell death counteracts exploitation of macrophage for *S*. Tm replication, it does not effectively kill the pathogen (Franchi et al. 2012). Instead, it traps *S*. Tm in cell debris and triggers the release of IL-1β (Franchi et al. 2012; Seo et al. 2015; Jorgensen et al. 2016). This IL-1β cytokine response recruits bactericidal neutrophils, which efferocytose the cell debris and kill the trapped *S*. Tm (Franchi et al. 2012; Jorgensen et al. 2016). Thus, while CX3CR1^hi^ macrophage pyroptosis does not directly kill *S*. Tm, it plays an essential role in the multi-layered defence by facilitating pathogen clearance through neutrophil action. This points to an intricate crosstalk between the pathogen's virulence factors and innate cell autonomous defences. J. Kagan has proposed the ”infection infidelity hypothesis”, which suggests that disease results from those rare events of pathogen-host cell encounters, where the pathogen fails to fully derail innate defence which permits the respective host cells to mount an appropriate inflammatory response (Kagan 2023). The interplay between *S*. Tm virulence factors and innate defence mechanisms in CX3CR1^hi^ macrophages suggests that the infection infidelity hypothesis may apply during *S*. Tm infection. Occasionally, *S*. Tm fails to fully subvert macrophage defences, allowing these cells to initiate pro-inflammatory responses. These responses promote pathogen clearance but also contribute to pathology, increasing the likelihood of symptomatic disease.

### Intestinal conventional dendritic cells

Intestinal cDCs are highly migratory cells that play a key role in the induction of oral tolerance (Luciani et al. 2022). These cells sample the gut lumen for food antigens, microbiota members and pathogens (Hapfelmeier et al. 2008; Schulz et al. 2009; Farache et al. 2013). They additionally receive antigens from goblet cells, macrophages, M cells and apoptotic IECs (Huang et al. 2000; Jang et al. 2004; McDole et al. 2012; Mazzini et al. 2014). This provides cDCs with a broad range of antigens necessary to establish oral tolerance. Upon antigen uptake, cDCs undergo maturation and upregulate CCR7 expression, facilitating their migration through the lymph to mLNs that drain specific regions of the GI tract (Worbs et al. 2006). In these mLNs, cDCs drive the priming of CD4^+^ and CD8^+^ T cells, which then home back to the specific GI regions from which the antigens were derived (Houston et al. 2016). Consequently, cDCs serve as a link between the innate and adaptive immune systems, aiding homeostasis of the multi-layered intestinal barrier.

Intestinal cDCs are characterised by a tolerogenic state that is imprinted by environmental factors, such as SCFAs, retinoic acid and TGF-β (Singh et al. 2014; Zeng et al. 2016; Bain et al. 2017). Two distinct cDC populations have been reported; CD11b^−^ CD103^+^ cDC1 and CD11b^+^ CD103^+/−^ cDC2. Within the cDC2 population, CD103^+^ cDC2 cells are predominant in the small intestine, while CD103^−^ cDC2 cells are more abundant in the large intestine (Bogunovic et al. 2009; Cerovic et al. 2013; Farache et al. 2013). The distribution of these cDC subsets throughout the GI tract depends on the microbiota density, composition and dietary factors. For example, variations in microbiota and diet across different mouse vendors and facilities affect the abundance of cDC subsets in different GI regions (Denning et al. 2011; Cerovic et al. 2013; Scott et al. 2015). Regardless of the subset, all cDCs can induce Tregs and contribute to oral tolerance through production of factors such as retinoic acid, TGF-β and IL-33 (Coombes et al. 2007; Sun et al. 2007; Esterházy et al. 2016; Hung et al. 2020). Intestinal cDC1s are most proficient in cross-presenting antigens to CD8^+^ T cells, whereas intestinal cDC2 cells are more effective at activating CD4^+^ T cells, such as Th17 cells (Den Haan, Lehar and Bevan 2000; Dudziak et al. 2007; Persson et al. 2013; Schlitzer et al. 2013). The implications of the differences in cDC subset functions in intestinal homeostasis are incompletely understood and require further investigation.

During *S*. Tm infection, TLR ligands and chemokines amplify the recruitment of cDCs into the intestinal tissue, enhancing their migratory capacity and promoting dendrite extension through the epithelial barrier, fostering sampling of gut-luminal *S*. Tm (Chieppa et al. 2006; Farache et al. 2013). Among the cDC subsets, both cDC1 and cDC2 subsets take up *S*. Tm in the lamina propria, whereas *S*. Tm-containing cDC2s primarily migrate to the mLN (Bogunovic et al. 2009; Carden et al. 2017). This migration is exploited by *S*. Tm to systemically disseminate, as demonstrated in *Flt3*^−/−^ mice, lacking DCs, and *Ccr7*^−/−^ mice, lacking the receptor required for cDC migration, which show a 5-fold reduction in *S*. Tm loads in the mLN compared to control mice at 2 days post infection (Hapfelmeier et al. 2008; Bogunovic et al. 2009; Kaiser et al. 2013, 2014). While this migration might be beneficial for *S*. Tm dissemination in the short term, it also facilitates antigen presentation in the mLN, which is essential for initiating adaptive immune responses. Specifically, cDCs employ controlled phagosome acidification that degrades *S*. Tm into peptides for cross-presentation on MHC-I molecules, initiating *S*. Tm-specific CD8^+^ T cell responses (Salazar-Gonzalez et al. 2006; Savina et al. 2006; Farache et al. 2013). These CD8^+^ T cell responses are particularly important to control *S*. Tm loads at systemic sites, which plays a key role in preventing progression into symptomatic disease (Lee et al. 2012). This highlights the role of intestinal cDCs in activating *S*. Tm-specific adaptive immune responses, underscoring their function in the multi-layered intestinal barrier.

### IgA production and vaccination strategies

Production of IgA by long-lived intestinal plasma B cells is a key component of the intestinal adaptive immune system that contributes to intestinal homeostasis. IgA, the predominant antibody in the intestinal lumen, is produced in response to the commensal microbiota upon TLR signalling and recognition of SCFAs (Slack et al. 2009; Hapfelmeier et al. 2010; Kim et al. 2016). Plasma B cells produce a diverse IgA repertoire, which coats a large fraction of the commensal microbiota (Hapfelmeier et al. 2010; Lindner et al. 2015). Interestingly, this IgA is polyreactive, it can bind weakly to multiple microbiota members and pathogens (Bunker et al. 2017; Rollenske et al. 2018). The diversity and broad reactivity are established in the germinal centres (GCs) of Peyer's patches where B cells undergo somatic hypermutations, which drive B cell receptor (BCR) affinity maturation against antigens expressed by commensal microbes (Chen et al. 2020; Nowosad et al. 2020). This IgA response is important in maintaining host-microbiota symbiosis. It not only protects against translocation of microbiota members and systemic dissemination, but also aids in clearing bacteria from the small intestine, limiting the penetration of microbiota-derived metabolites into host tissues (Uchimura et al. 2018). Through these mechanisms, IgA production shapes the composition and diversity of the microbiota, thereby contributing to the homeostasis of the multi-layered intestinal barrier (Slack et al. 2009; Kawamoto et al. 2014).

IgA production also plays a protective role in *S*. Tm infection. The host can raise IgA specific to *S*. Tm LPS O-antigens, which restricts gut luminal *S*. Tm growth and prevents contact with the epithelial barrier (Endt et al. 2010). This finding has laid the foundation for vaccination strategies that stimulate *S*. Tm-specific IgA responses. Oral vaccination against *S*. Tm drives the production of *S*. Tm-specific IgA, which binds not only to *S*. Tm but also to its daughter cells upon division, leading to enchained growth (Moor et al. 2017). This results in clumping of clonal *S*. Tm, thereby enhancing their clearance from the gut lumen and providing a robust defence against infection (Moor et al. 2017). This IgA response exerts selective pressure on *S*. Tm, favouring strains with shorter O-antigen chains that evade IgA recognition (Diard et al. 2021). These shorter O-antigen variants are more susceptible to other antimicrobial defences and are therefore less likely to cause disease (Diard et al. 2021; Gül et al. 2023a). Importantly, the frequency of such O-antigen mutants among natural *Salmonella* isolates suggests that sIgA-mediated enchained growth is relevant also in natural infections (Cherry and Eyre-Walker 2020). Thus, sIgA responses create a trade-off, which the pathogen cannot easily circumvent. These findings suggest that vaccination strategies that promote IgA responses could serve as effective prophylactic measures against *S*. Tm infections. As farm animals are natural carriers of *S*. Tm and transmission to humans often occurs through contaminated food, targeting farm animals with such vaccination strategies may be particularly beneficial in limiting human symptomatic *S*. Tm infection (Lentsch et al. 2023).

Collectively, the immune cell types described above form an important layer of the multi-layered defence against *S*. Tm infection by employing diverse protective mechanisms, including ROS-mediated bacterial killing, pro-inflammatory cytokine release, induction of *S*. Tm-specific adaptive immune responses, and IgA-mediated gut-luminal clearance. Whether *S*. Tm exposure results in asymptomatic carriage or symptomatic illness is strongly influenced by inter-individual variation in the immune system (Crump et al. 2015; Gilchrist et al. 2015). Immunocompromised patients are particularly at risk of *S*. Tm-induced disease, as conditions such as NADPH oxidase dysfunction in phagocytes, deficiencies in cytokines IL-12 or IL-23, and deregulated humoral immunity in HIV-infected individuals are associated with increased susceptibility to *S*. Tm infection (De Jong et al. 1998; MacLennan et al. 2004, 2010; van den Berg et al. 2009; Gilchrist et al. 2015). Beyond immunodeficiency, genetic polymorphisms and microbiota composition also shape antimicrobial functions of immune cells. Specifically, polymorphisms in pathways downstream of TLRs, including MyD88 and IκBα, can dampen antimicrobial responses, thereby increasing susceptibility to disease (Picard et al. 2011). Thus, the likelihood of symptomatic disease following *S*. Tm exposure depends on immune deficiencies, genetic predisposition, and microbiota composition, which collectively determine the host's antimicrobial capacity.

## Mechanisms used by *S*. Tm to overcome the multi-layered host protection

We will next explore the specific mechanisms and adaptations employed by *S*. Tm to overcome the host's multi-layered defence mechanisms and how this impacts the outcome of infection. Central to these mechanisms is the ability to trigger and exploit intestinal inflammation (Stecher et al. 2007). In the gut lumen, *S*. Tm uses its flagellin-mediated motility and the T3SS-1 to reach and invade IECs, which initiates intestinal inflammation (Hapfelmeier et al. 2004; Stecher et al. 2004, 2007, 2008). This inflammation is associated with release of gut-luminal nutrients and terminal electron acceptors, along with the production of AMPs and ROS, creating an antimicrobial environment (Müller et al. 2009; Stelter et al. 2011; Felmy et al. 2013). While these changes negatively impact the microbiota, *S*. Tm has evolved mechanisms to exploit this environment, providing *S*. Tm with a competitive advantage over the microbiota (Lupp et al. 2007; Stecher et al. 2007; Kröger et al. 2013). In the tissue, *S*. Tm employs a distinct set of mechanisms, including T3SS-2-mediated pathways to evade host immune responses, establish a replicative niche, and disseminate systemically (Müller et al. 2012; Chen et al. 2021a). By tailoring its virulence mechanisms to distinct environments within the host, *S*. Tm effectively exploits and overcomes intestinal defences. Understanding the mechanisms underlying virulence sheds light on how *S*. Tm can leverage multi-layered intestinal defence mechanisms to successfully infect the host.

### Utilisation of liberated nutrients and liberated terminal electron acceptors

**Figure 7.**
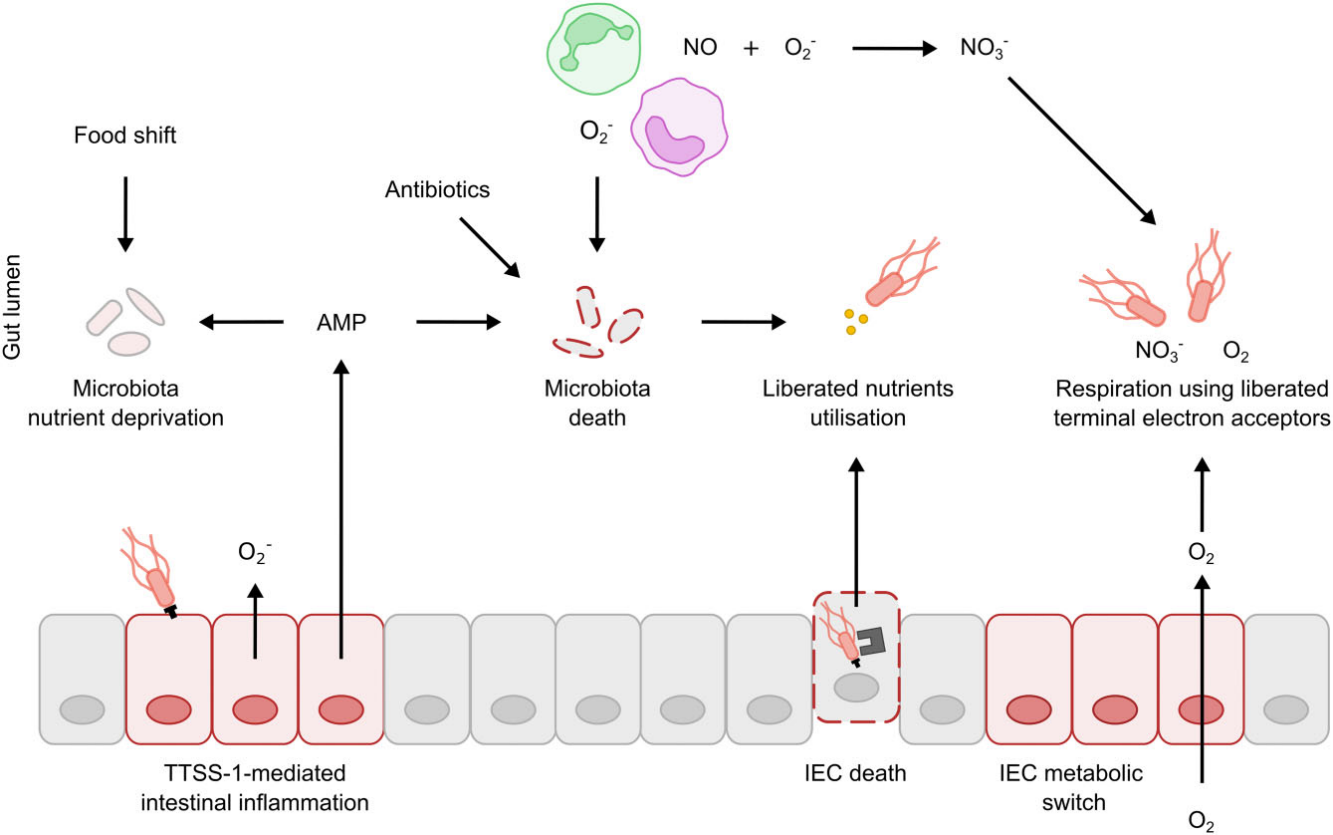
Mechanisms used by *S*. Tm to overcome the intestinal multi-layered barrier. *S*. Tm employs various mechanisms to fuel gut-luminal colonisation, particularly upon environmental changes and intestinal inflammation. These conditions disrupt colonisation resistance by inducing microbiota and IEC death, releasing nutrients that *S*. Tm can exploit. Additionally, antimicrobial peptides (AMPs) and food shifts deprive the microbiota of essential nutrients, thereby reducing the production of inhibitory metabolites. This inflammatory environment further liberates terminal electron acceptors, like oxygen and nitrate, which *S*. Tm can exploit for respiration to outcompete the microbiota.

Under homeostatic conditions, the microbiota efficiently exploits nutrients, limiting their availability in the gut lumen (Zeng et al. 2022; Meier et al. 2023). However, disruptions such as inflammation-induced IEC death liberate nutrients into the intestinal lumen (Anderson et al. 2021) (Fig. 7). In a similar manner, microbiota cell death caused by antibiotic treatment or inflammation releases intracellular nutrients, alters microbiota composition and halts gut-luminal nutrient exploitation by the microbiota (Ng et al. 2013; Yoo et al. 2024). These disruptions open nutrient niches that can be exploited by *S*. Tm. When sensing these liberated nutrients, including amino acids and carbohydrates, *S*. Tm upregulates metabolic pathways to fuel colonisation of these opened niches (Ng et al. 2013; Kitamoto et al. 2020; Yoo et al. 2024). For instance, *S*. Tm utilises aspartate from lysed microbiota cells to fuel its growth (Yoo et al. 2024). This metabolic flexibility demonstrates how *S*. Tm exploits nutrient perturbations to overcome colonisation resistance, gaining a competitive advantage over the microbiota in the perturbed gut environment.

Microbiota perturbations also influence the availability of terminal electron acceptors in the gut lumen. Under homeostasis, gut-luminal oxygen pressure decreases from ∼6% in the small intestine to ∼0.6% in the large intestine (Albenberg et al. 2014). Large intestinal IECs utilise β-oxidation to oxidise microbiota-derived butyrate, fuelling ATP synthesis (Roediger 1982). This process consumes oxygen that diffuses from the circulation into the tissue, and thereby controls gut-luminal oxygen availability, driving the microbiota to rely on anaerobic fermentation (Kelly et al. 2015). However, the use of antibiotics depletes butyrate-producing microbiota members, switching IEC metabolism from β-oxidation to lactate fermentation, leading to diffusion of excess oxygen into the gut lumen (Kelly et al. 2015; Rivera-Chávez et al. 2016b). This creates a niche that facultative anaerobes can exploit for aerobic respiration (Rivera-Chávez et al. 2016b). For example, *S*. Tm utilises cytochrome *bd*-II oxidase (CyxAB) to use oxygen as the terminal electron acceptor in the electron transport chain, facilitating ATP generation in the oxygenated gut of antibiotic-pretreated mice (Rivera-Chávez et al. 2016b). This flexibility to switch to aerobic respiration, which most microbiota members lack, enables *S*. Tm to successfully compete and grow after microbiota perturbations.

Inflammatory responses further augment terminal electron acceptor availability. Recognition of *S*. Tm triggers the production of superoxide (O_2_^−^) by IECs through NADPH oxidase 1 (NOX1) and by neutrophils and monocytes through NOX2. Additionally, both IECs and innate immune cells produce nitric oxide (NO) via iNOS. During *S*. Tm infection, neutrophil-derived nitric oxide reacts with superoxide to form nitrate (NO_3_^−^) in the gut lumen, which *S*. Tm utilises through nitrate reductase complexes NarGHJI NarZYWV NapFDAGHBC to facilitate ATP generation in the inflamed gut (Lopez et al. 2012; Winter et al. 2013; Liou et al. 2022). Additionally, the gut microbiota produces hydrogen sulphide (H_2_S), which is detoxified into thiosulphate (S_2_O_3_^2−^) by the intestinal tissue (Levitt et al. 1999). Under intestinal inflammation, thiosulphate reacts with superoxide, forming tetrathionate (S_4_O_6_^2−^) which *S*. Tm can use for tetrathionate respiration through tetrathionate reductase enzyme complexes TtrSR TtrBCA (Winter et al. 2010). Most microbiota members lack nitrate and tetrathionate reductase enzyme complexes, which provide *S*. Tm with an advantage over the microbiota in the inflamed gut (Lopez et al. 2012). Thus, *S*. Tm can exploit host defence mechanisms to fuel colonisation of the gut lumen.

The central metabolism of *S*. Tm is also altered by increased nutrient and terminal electron acceptor availability following microbiota perturbations and inflammation (Spiga et al. 2017; Nguyen et al. 2024). Under homeostasis, when terminal electron acceptor availability is limited, the TCA cycle of *S*. Tm is split and operates partially in reverse orientation, catalysing the reduction of fumarate into succinate via fumarate reductase (Frd) (Spiga et al. 2017; Nguyen et al. 2020). When terminal electron acceptors are liberated, *S*. Tm switches to the oxidative TCA cycle, in which succinate dehydrogenase (Sdh) oxidises succinate into fumarate. The oxidative TCA cycle is beneficial to *S*. Tm as it yields more ATP than the reverse TCA cycle (Spiga et al. 2017; Nguyen et al. 2020). Recent findings further indicate that *S*. Tm adapts its mixed-acid fermentation pathways during gut colonisation, initially relying on acetate fermentation and fumarate respiration, while ethanol fermentation and α-ketoglutarate production become more prominent at the onset of gut inflammation (Nguyen et al. 2024). This helps to balance redox states and conserve energy in response to changes in nutrient and terminal electron acceptor availability, thereby enhancing the ability of the pathogen to achieve higher gut-luminal densities (Nguyen et al. 2024). *S*. Tm can additionally remodel the intestinal environment through mixed acid fermentation by depleting microbiota-derived SCFAs and increasing epithelial oxygenation, creating a niche that favours *S*. Tm over obligate anaerobes (Rogers et al. 2024). Notably, these changes occur before detectable shifts in microbiota composition, indicating that engineering of the gut environment is one of several mechanisms through which *S*. Tm can break colonisation resistance (Rogers et al. 2024). This metabolic flexibility together with its capacity to reshape host-microbiota interactions, provides *S*. Tm with a competitive advantage that supports high-density gut-luminal colonisation. Collectively, this underscores how microbiota perturbations, intestinal inflammation, and pathogen-driven environmental changes contribute to the ability of *S*. Tm to exploit liberated nutrient and terminal electron acceptors to fuel gut-luminal growth.

### Resistance to antimicrobial peptides

*S*. Tm-induced intestinal inflammation also triggers the release of AMPs into the gut lumen. TLR5 mediated recognition of flagellin by cDCs triggers the release of IL-23 (Kinnebrew et al. 2012). IL-23 prompts the release of IL-22 by ILCs and T cells, which boosts the production of AMPs, such as lipocalin-2, calprotectin, Reg-IIIβ and Reg-IIIγ, by IECs and neutrophils (Kinnebrew et al. 2012; Liu et al. 2012; Behnsen et al. 2014; Loonen et al. 2014). These AMPs either sequester essential nutrients, limiting their availability to pathogens in the gut lumen, or act as bactericidal agents. For example, lipocalin-2 sequesters enterochelin, a siderophore that enables iron acquisition, to limit iron availability (Raffatellu et al. 2009; Behnsen et al. 2014). However, *S*. Tm can utilise IroBCDE IroN for the biosynthesis and uptake of salmochelin, a siderophore not sequestered by lipocalin-2 (Raffatellu et al. 2009; Behnsen et al. 2014). This provides *S*. Tm with a competitive advantage over commensal microbiota members that rely solely on enterochelin for iron uptake (Raffatellu et al. 2009; Behnsen et al. 2014). Notably, some commensal bacteria, such as *Bacteroides thetaiotaomicron*, have adapted by encoding a xenosiderophore utilisation system (XusABC), that binds enterochelin, protecting it from lipocalin-2 sequestration and facilitating iron uptake (Zhu et al. 2020; Spiga et al. 2023). *S*. Tm can also utilise this XusB-bound enterochelin for iron acquisition, demonstrating its ability to employ multiple mechanisms to evade iron limitation (Spiga et al. 2023). In a similar manner, calprotectin sequesters metal ions, including zinc and manganese, which are essential for bacterial growth (Liu et al. 2012; Behnsen et al. 2014; Diaz-Ochoa et al. 2016). *S*. Tm overcomes this by employing the high-affinity zinc transporter ZnuABC and manganese transporters SitA and MntH, enabling it to overcome zinc and manganese sequestration by calprotectin and gain a competitive edge over the microbiota (Liu et al. 2012; Behnsen et al. 2014; Diaz-Ochoa et al. 2016). Another class of AMPs, including Reg-IIIβ and Reg-IIIγ, act as bactericidal lectins, permeabilising the outer membrane of bacteria and subsequently triggering bacterial death (Miki and Hardt 2013; Mukherjee et al. 2014). Prominent microbiota members, such as *Bacteroides* spp., are susceptible to Reg-IIIβ while *S*. Tm is not, resulting in a loss of these microbiota members and compromising colonisation resistance against *S*. Tm (Stelter et al. 2011; Miki et al. 2017). Thus, while the host aims to restrict pathogen growth through AMPs, *S*. Tm has evolved mechanisms to overcome these limitations, facilitating *S*. Tm colonisation by providing it with a competitive advantage over the microbiota.

### Virulence selection

**Figure 8.**
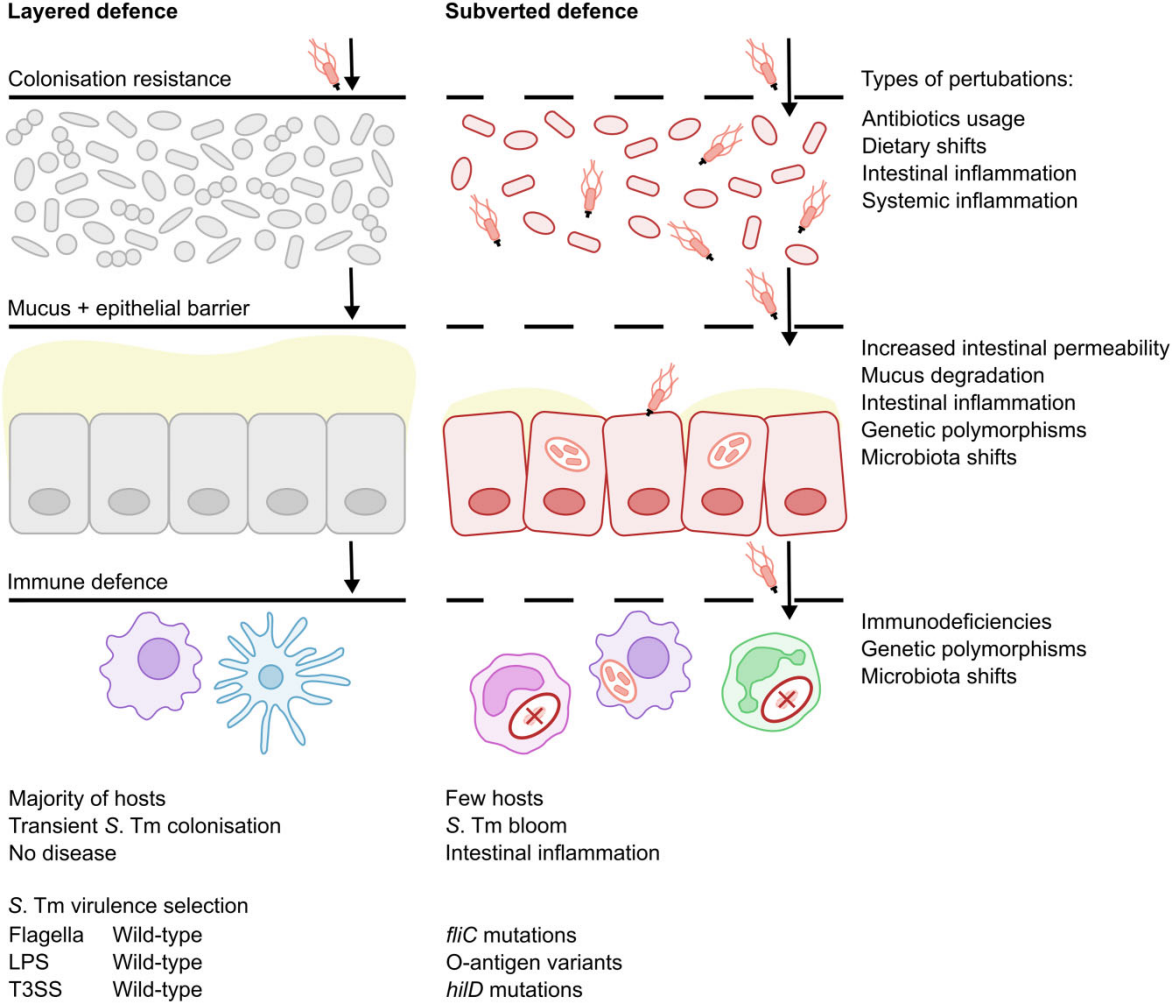
Two fundamentally different gut-luminal environments for *S*. Tm shaped by host defences and perturbations. The microbiota, epithelial barrier, and immune system collectively provide a multi-layered defence that limits *S*. Tm to transient colonisation and prevents disease. However, disruptions in these defences can allow *S*. Tm to proliferate in the gut lumen and trigger intestinal inflammation. Key perturbations include: microbiota disruptions (e.g. antibiotic use, dietary shifts, intestinal or systemic inflammation), epithelial barrier impairment (e.g. increased intestinal permeability, mucus degradation, inflammation, genetic polymorphisms, microbiota shifts), and immune system dysfunction (e.g. immunodeficiencies, genetic polymorphisms, microbiota shifts). These conditions often drive selection for *S*. Tm virulence adaptations, such as *fliC* and *hilD* mutations and O-antigen variants, which reduce virulence while fostering persistence and proliferation.

The ability of *S*. Tm to adapt to distinct gut environments plays a critical role in determining infection outcomes (Fig. 8). In most hosts with intact multi-layered defences, *S*. Tm persists at low densities within a microbiota-dominated, nutrient-limited environment. In this context, *S*. Tm utilises mixed acid fermentation of scarce carbohydrates and other available nutrients (Nguyen et al. 2020, 2024; Schubert et al. 2025b). Interestingly, this microbiota-dominated niche selects for classical virulence factors including flagella for motility, LPS for protection against AMPs, bile acids and complement, and T3SS expression for host cell invasion (Cherry and Eyre-Walker 2020; Gül et al. 2023a). However, perturbations of the multi-layered defence, characterised by microbiota dysbiosis and virulence-induced inflammation, create an inflammatory niche where selective pressures disrupt the microbiota and favour *S*. Tm mutants with attenuated virulence (Cherry and Eyre-Walker 2020; Gül et al. 2023a; Grote et al. 2024). In this environment, mutations in *fliC* reduce immune recognition through flagellin, shortened O-antigens help evade antibody recognition, and mutations in *hilD* counteract the energy cost associated with T3SS-1 expression (Cherry and Eyre-Walker 2020; Gül et al. 2023a). These adaptations of *S*. Tm to the inflammatory environment enhance immune evasion and fitness, linking symptomatic disease to perturbed niches that select for less virulent but more persistent strains (Grote et al. 2024).

### Invasive *S*. Tm and adaptations to bloodstream infections

While much of our understanding of *S*. Tm infection focuses on gut-luminal colonisation and inflammation-driven nutrient exploitation, certain lineages have evolved to thrive in fundamentally different host environments (Perez-Sepulveda and Hinton 2025). A striking example is the *S*. Tm ST313 lineage, which has emerged in sub-Saharan Africa as a major cause of invasive non-typhoidal salmonellosis (iNTS) (Pulford et al. 2021). iNTS is characterised by bloodstream infection and disproportionately affects immunocompromised individuals, including those with HIV or malaria. Unlike the globally prevalent ST19 lineage, which typically causes self-limiting gastroenteritis, ST313 infections often occur without gastrointestinal symptoms (Ramachandran et al. 2017). Genomic analyses have revealed that ST313 differs from ST19 in several key features, particularly in genes related to metabolism and virulence regulation (Ramachandran et al. 2015; Canals et al. 2019). These genomic signatures have been interpreted as signs of host adaptation, although the exact functional consequences remain to be fully defined. The high prevalence of immunocompromised hosts in sub-Saharan Africa likely provided a permissive niche for the emergence and spread of ST313. Other region-specific factors, such as dietary habits, microbiota composition, and environmental exposures, may also have shaped its evolution. Ongoing *S*. Tm challenge studies comparing ST313 and ST19 will further assess ST313-specific virulence (Smith et al. 2024). These adaptations exemplify how *S*. Tm can escape gut-associated selective pressures and evolve to exploit distinct ecological and immunological contexts, bypassing multi-layered intestinal defences and infecting systemic sites.

Collectively, the ability of *S*. Tm to successfully infect the host reflects a dynamic balance between metabolic adaptability and virulence factors to exploit perturbations in the multi-layered barrier. Virulence factors drive inflammatory responses that facilitate gut-luminal *S*. Tm colonisation through exploitation of liberated nutrients and terminal electron acceptors. However, maintaining virulence imposes a metabolic cost and increases host immune activation, driving selection for attenuated strains that are better at persisting and immune evasion (Cherry and Eyre-Walker 2020; Gül et al. 2023a; Grote et al. 2024). To mitigate selection pressures that favour loss of virulence factors, virulence-mediated tissue invasion fosters the formation of tissue reservoirs that escape gut-luminal selection pressures, enabling *S*. Tm to retain virulence factors and reseed into the gut lumen when conditions become favourable (Diard et al. 2013). This underscores the multi-faceted mechanisms that *S*. Tm utilises to overcome the multi-layered intestinal protection. Consequently, the likelihood of symptomatic disease versus asymptomatic carriage depends on the specific *S*. Tm strain and its combined virulence and metabolic capabilities. More virulent and metabolically adept strains are more likely to trigger strong inflammatory responses, create favourable conditions for gut-luminal colonisation and tissue invasion, leading to symptomatic disease.

## Current limitations and future directions in *S*. Tm research

Murine models have been indispensable for uncovering fundamental mechanisms of *S*. Tm pathogenesis. They offer experimental control and have provided deep insights into how *S*. Tm breaches host barriers, manipulates immune responses, and competes within the intestinal environment. Nonetheless, translating these findings to humans remains challenging. Anatomical and physiological differences, particularly in the gastrointestinal tract, can influence infection dynamics (Nguyen et al. 2015). For example, mice do not typically develop diarrhoea following *S*. Tm infection, indicating species-specific differences in immune activation and disease progression. Similarly, microbiota composition and function differ markedly between mice and humans (Nguyen et al. 2015; Beresford-Jones et al. 2022). These limitations are further amplified in simplified systems such as germ-free or gnotobiotic mice, where altered caecal morphology and impaired immune development may distort physiological responses (De Agüero et al. 2016; Hoces et al. 2022). To help bridge these translational gaps, human-relevant model systems are increasingly being adopted. Organoids derived from human intestinal tissue preserve key aspects of epithelial biology and immune signalling, enabling mechanistic studies in a species-specific context. Likewise, mice colonised with human-derived microbiota offer a tractable platform to dissect host–microbe–pathogen interactions under more physiologically relevant conditions. These models complement traditional murine approaches by allowing validation of human-specific findings and improving our ability to study the context-dependent dynamics of *S*. Tm infection.

Our understanding of asymptomatic *S*. Tm infections in humans remains incomplete, largely because most current evidence is based on indirect serological data. Seroconversion may reflect past exposure rather than active infection, as it can occur even after ingestion of non-viable bacteria from properly cooked, contaminated food. This complicates the interpretation of seroprevalence studies and obscures the true incidence and immunological relevance of asymptomatic carriage. To address these limitations, human challenge studies provide a powerful and increasingly important tool (Smith et al. 2024). By administering defined doses of *S*. Tm to healthy volunteers under controlled clinical conditions, these studies enable real-time, causally interpretable assessment of infection dynamics, immune correlates of protection, and the frequency of asymptomatic outcomes. Unlike retrospective or serological data, they allow for precise control over exposure and follow-up. Future work should aim to expand these studies across more diverse populations, incorporating standardised clinical endpoints and extended monitoring to better understand host variability in disease susceptibility.

Advancing targeted strategies for *S*. Tm control will require both improved diagnostics and interventions that support host and microbiota resilience. Non-invasive biomarkers, such as faecal metabolite profiles, offer a promising approach to detect functional shifts in the microbiota that may signal increased infection risk (Lehmann et al. 2024). Incorporating such tools into clinical settings could enable early risk stratification and guide personalised prevention or treatment strategies. In parallel, microbiota-directed therapies, including probiotics, prebiotics, and diet-based interventions, hold potential to restore colonisation resistance and reinforce mucosal immunity in at-risk individuals. At the population level, livestock vaccination against *S*. Tm remains a powerful measure to reduce pathogen prevalence in the food supply and limit human exposure (Lentsch et al. 2023, 2025). Taken together, these approaches provide a foundation for aligning mechanistic insights with clinical relevance, moving the field toward predictive and personalised strategies for *S*. Tm infection control.

## Concluding remarks

The outcome of *S*. Tm exposure is shaped by a complex interplay between host defences and pathogen adaptations. The intestinal microbiota, epithelial barrier, and immune system each contribute distinct mechanisms to the multi-layered protection that restricts infection and maintains gut homeostasis. Variability in the composition and function of these layers, driven by factors such as genetics, comorbidities, developmental stage, and environmental perturbations, can alter susceptibility to disease. At the same time, *S*. Tm has adapted its virulence and metabolism to exploit disruptions in the protective layers, facilitating colonisation, invasion, and immune evasion. While the precise contribution of each defence layer to protection remains difficult to quantify, current evidence suggests that the intestinal microbiota may play a particularly influential role in limiting *S*. Tm colonisation and expansion, potentially acting as a first line of defence. Mucosal immunity and barrier integrity likely act in concert with the microbiota, contributing additional layers of protection through physical separation, antimicrobial responses, and immune regulation. However, it is important to note that the anatomy of the murine caecum, which is the primary site of infection in murine *S*. Tm infection, differs substantially from the human gut (Nguyen et al. 2015). Thus, in the absence of comprehensive comparative data, these observations derived from mouse infection experiments remain speculative and should be interpreted with caution. Moving forward, defining how these layers of defence interact and how their disruption tips the balance from asymptomatic colonisation to disease will be critical for guiding targeted prevention and treatment strategies in vulnerable populations.
